# In Situ Determination of Structure and Fluctuations of Coexisting Fluid Membrane Domains

**DOI:** 10.1016/j.bpj.2014.11.3488

**Published:** 2015-02-17

**Authors:** Peter Heftberger, Benjamin Kollmitzer, Alexander A. Rieder, Heinz Amenitsch, Georg Pabst

**Affiliations:** 1 Biophysics Division, Institute of Molecular Biosciences, University of Graz, NAWI Graz, Graz, Austria; 2 BioTechMed-Graz, Graz, Austria; 3 Institute of Inorganic Chemistry, Graz University of Technology, Graz, Austria

## Abstract

Biophysical understanding of membrane domains requires accurate knowledge of their structural details and elasticity. We report on a global small angle x-ray scattering data analysis technique for coexisting liquid-ordered (*L*_*o*_) and liquid-disordered (*L*_*d*_) domains in fully hydrated multilamellar vesicles. This enabled their detailed analysis for differences in membrane thickness, area per lipid, hydrocarbon chain length, and bending fluctuation as demonstrated for two ternary mixtures (DOPC/DSPC/CHOL and DOPC/DPPC/CHOL) at different cholesterol concentrations. *L*_*o*_ domains were found to be ∼10 Å thicker, and laterally up to 20 Å^2^/lipid more condensed than *L*_*d*_ domains. Their bending fluctuations were also reduced by ∼65%. Increase of cholesterol concentration caused significant changes in structural properties of *L*_*d*_, while its influence on *L*_*o*_ properties was marginal. We further observed that temperature-induced melting of *L*_*o*_ domains is associated with a diffusion of cholesterol to *L*_*d*_ domains and controlled by *L*_*o*_/*L*_*d*_ thickness differences.

## Introduction

Since the formulation of the raft model in 1997 by Simons and Ikonen (1) significant scientific efforts have been devoted to the characterization of physical properties of liquid-disordered *L*_*d*_ and liquid-ordered *L*_*o*_ domains (2–11). Membrane rafts are thought to be small (nanoscopic) and highly dynamic platforms enriched in sphingolipids and cholesterol, enabling diverse cellular functions, but have so far escaped any direct visualization in live cells (5,12). Hence, the existence of rafts remains a highly controversial issue. For example Frisz et al. (13,14), using secondary ion mass spectrometry on fibroblasts, observed sphingolipid domains, in which cholesterol was evenly distributed throughout the membrane, thus challenging the standard raft hypotheses.

In contrast to natural membranes, domains in lipid-only systems can grow up to several micrometers in size, enabling their detection (e.g., by optical microscopy (15)) and study with respect to the physics pertaining to their stability, size, or effect on protein sorting, to name but a few examples (8). One of the parameters involved in, e.g., protein sorting, is the difference in thickness between the *L*_*o*_ and *L*_*d*_ domains and the corresponding match to the protein’s transmembrane region (see, e.g., Killian (16) and Pabst (17)).

To address these issues, diverse experimental and theoretical techniques have been employed to explore structural and elastic properties of *L*_*o*_/*L*_*d*_ phases (see, e.g., the literature (18–32)). Scattering experiments are of particular interest in this respect, because they allow for a label-free determination of membrane structure and dynamics (33). However, contrast between *L*_*o*_ and *L*_*d*_ domains is low. This can be addressed, for example, by contrast variation, using neutron scattering (34). In recent years, this technique has been used largely by Katsaras and coworkers, showing, e.g., the coupling of domain size and membrane thickness mismatch between *L*_*o*_ and *L*_*d*_ (35).

Alternatively, early x-ray experiments used Triton X-100 (Dow Chemical, Midland, MI) to separate detergent-resistant from detergent-soluble membranes, respectively (22). However, the application of detergents on membranes may adversely influence the mixing behavior of membrane lipids (36), limiting the applicability of this approach.

Another possibility, which is being explored in this work, makes use of the experimental finding that macroscopic domains are typically in registry in multilamellar systems (see, e.g., Chen et al. (28), Tayebi et al. (37), and Karmakar et al. (38)), meaning: *L*_*o*_ and *L*_*d*_ domains form lamellar lattices with distinct Bragg peaks. The challenges to be met here are 1) overlapping *L*_*o*_/*L*_*d*_ Bragg reflections, in particular at low scattering angles; and 2) the small number of solid orders (only 2–3) displayed by *L*_*d*_ phases in fully hydrated multilamellar vesicles (MLVs), limiting the structural information content when only Bragg peak intensities are analyzed (39).

The latter issue is particularly well known for single-phase fluid bilayers, and has led to the development of a global SAXS data analysis technique that takes into account both Bragg peaks and diffuse scattering (39). Most recently, we have advanced the technique by incorporating the scattering density profile (SDP) model (40), enabling us to determine membrane structure and bending fluctuations from homogeneous MLVs at high resolution (41).

**Figure 1:**
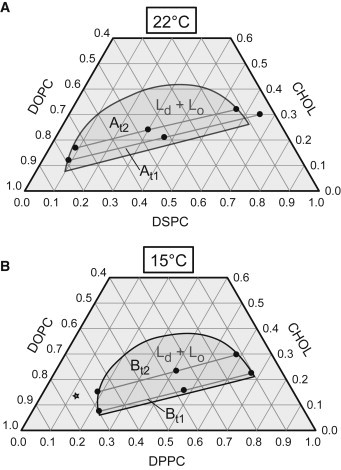
Overview of samples studied in this work. (*Solid circles*) Their location is shown in previously reported compositional phase diagrams of (*A*) DOPC/DSPC/CHOL (50) and (*B*) DOPC/DPPC/CHOL (49). In fluid-fluid phase coexistence regions (*dark-shaded areas*), demixing into *L*_*o*_ and *L*_*d*_ domains occurs along tielines. Two tielines for each system (*A*_*t*1_, *A*_*t*2_ and *B*_*t*1_, *B*_*t*2_) with three different compositions at the *L*_*d*_, *L*_*o*_ endpoints and the tieline center were studied (see Table S1 for detailed lipid composition). Note that tieline endpoints for *L*_*o*_ on *A*_*t*1_ and *L*_*d*_ on *B*_*t*2_ (*star*) are outside previously reported phase boundaries. This is either due to updates in phase boundaries for isolated tielines (*A*_*t*1_ (35)) or experiments performed in this study (*B*_*t*2_, see Results).

To access coexisting fluid domains in MLVs, the global SAXS data analysis needs to be further extended. This was achieved in this work by assuming a linear combination of scattering intensities originating from *L*_*o*_ and *L*_*d*_ phases. The method was applied to two ternary mixtures, with the high-melting lipids DPPC (dipalmitoylphosphatidylcholine) or DSPC (distearoylphosphatidylcholine), the low-melting lipid DOPC (dioleoylphosphatidylcholine), and CHOL (cholesterol). Summaries of the studied samples and applied nomenclature are given in Fig. 1 and Table S1 in the Supporting Material.

We observed distinct structural and elastic properties of *L*_*o*_ and *L*_*d*_ domains as a function of temperature and composition (lipid chain length and cholesterol concentration). Most interestingly, we found that increased cholesterol concentrations reduce the thickness difference between *L*_*d*_ and *L*_*o*_ domains, which leads to a decrease of line tension and in turn promotes the temperature induced melting of *L*_*o*_ domains.

## Materials and Methods

### Sample preparation

DPPC, DSPC, and DOPC were purchased from Avanti Polar Lipids (Alabaster, AL), and cholesterol was obtained from Sigma-Aldrich (Vienna, Austria). All lipids were used without further purification, with all chemicals being of professional analysis quality. Lipid stock solutions were prepared by dissolving weighted amounts of dry lipid in chloroform/methanol (2:1, v/v) and then mixed at appropriate ratios (see Table S1 for all samples and their corresponding compositions). Subsequently, lipid solutions were dried under a stream of nitrogen and then placed under vacuum for ∼12 h, forming a thin lipid film on the bottom of glass vials. Dry films were hydrated using 18 MΩ/cm water and incubated for 6 h above the main transition temperature of the high-melting lipid (DPPC or DSPC), repetitively cycling vortex-mixing and freeze-thaw procedures using liquid N_2_. The final lipid concentration for each sample was 50 mg/mL. All samples were prepared at least twice (with a time delay of several weeks) starting from pure lipid powders to check for reproducibility. Lattice constants (*d*-values) varied by <±0.5%. Furthermore, thin layer chromatography on randomly selected samples revealed no decomposition of the samples into lyso lipids or free fatty acids.

### Small angle x-ray scattering

X-ray scattering data were acquired at the Austrian SAXS beamline Elettra Trieste, Trieste, Italy, using 8 keV photons. Diffraction profiles were detected utilizing a Mar300-image-plate detector (MarResearch, Norderstedt, Germany) and calibrated using silver behenate. Lipid dispersions were taken up in 1-mm-thick quartz capillaries and inserted into a multiposition sample holder. All samples were equilibrated for a minimum of 10 min before measurement using a circulating water bath. The exposure time was set to 30 s. Scattering patterns were integrated using the program FIT2D (42). Background scattering originating from capillaries, water, and air was subtracted (43), and data sets were normalized using the transmitted intensity, which was measured by a photodiode placed in the beam stop.

### Analysis of coexisting domains

To analyze the scattering profile of MLVs exhibiting *L*_*o*_/*L*_*d*_ phase coexistence, we adopted the full-*q*-range model by Heftberger et al. (41) for homogeneous bilayers. For the latter systems, the scattered intensity is given by

(1) $$I\left(q\right)=\frac{1}{q^{2}}\left[{\left|F\left(q\right)\right|}^{2}S\left(q\right)\left(1-N_{\text{diff}}\right)+\left|F{\left(q\right)}^{2}\right|N_{\text{diff}}\right],$$

where *q* = 4*π*sin*θ*/*λ* is the scattering vector, *λ* is the wavelength, 2*θ* is the scattering angle relative to the incident beam, and *N*_diff_ is the diffuse scattering originating from positionally uncorrelated bilayers. The structure factor *S*(*q*) is given by the Caillé theory (39,44–46), yielding access to bending fluctuations via the Caillé parameter

$$\eta \propto \frac{T}{\sqrt{K_{C}B}},$$

with *K*_*C*_ as the bilayer bending rigidity and *B* as the bulk modulus of interactions (39). The form factor *F*(*q*) is the Fourier transform of the electron density profile of a bilayer, described in terms of the SDP model (40).

Neglecting putative cross-correlations between *L*_*o*_ and *L*_*d*_ domains, the scattered intensity of coexisting fluid domains can be modeled by a linear combination of the individual *L*_*o*_/*L*_*d*_ intensities,

(2) $$I\left(q\right)=\frac{1}{q^{2}}\left[\left(1-N_{\text{diff}}\right)I_{\text{corr}}\left(q\right)+N_{\text{diff}}I_{\text{uncorr}}\left(q\right)\right],$$

where

$$I_{\text{corr}}\left(q\right)={\left|F_{Ld}\left(q\right)\right|}^{2}S_{Ld}\left(q\right)c_{Ld}+{\left|F_{Lo}\left(q\right)\right|}^{2}S_{Lo}\left(q\right)\left(1-c_{Ld}\right)$$

and

$$I_{\text{uncorr}}\left(q\right)={\left|F_{Ld}\left(q\right)\right|}^{2}c_{Ld}+{\left|F_{Lo}\left(q\right)\right|}^{2}\left(1-c_{Ld}\right)$$

are the scattering intensities originating from positionally correlated and uncorrelated bilayers, respectively. The subscripts *Lo* and *Ld* denote the individual contributions of the domains to *S*(*q*) and *F*(*q*), and *c_Ld_* refers to the *L*_*d*_ phase fraction in the sample. Analysis of the scattered intensity of coexisting phases in terms of this model yields bilayer structural parameters and bending fluctuations simultaneously for *L*_*o*_ and *L*_*d*_. A strict requirement for its application is that domains are in registry in the direction normal to the bilayer plane, meaning: two distinct lamellar lattices need to be observed. This is typically the case for macroscopic domains, observed for example when diunsaturated lipids such as DOPC are used as low-melting membrane component in ternary raftlike mixtures (28,37,38).

The SDP model, used to describe the form factors, parses the bilayer lipids into quasi-molecular fragments and calculates their volume probability distributions. The model was originally designed for determining single lipid component bilayers (40,47). Pan et al. (48) extended the SDP analysis to binary lipid mixtures containing cholesterol. To this end the contribution of cholesterol was merged with that of methylene (CH_2_) groups, which was recently also applied successfully to homogenous MLVs (41). Because our studied *L*_*o*_ and *L*_*d*_ domains contain different amounts of three lipids, effective lipid molecules were constructed for the SDP description, by first merging the contributions from the unsaturated and saturated lipids and then adding cholesterol to the CH_2_ regime as described above. Saturated and unsaturated lipids differ with respect to the number of CH_2_ and methine (CH) groups. Due to the absence of scattering contrast between CH and CH_2_ for x-rays, these groups can be merged (40,41). Our final parsing approach consisted of five groups for each phase, composed of the following: 1) CholCH_3_ (Choline methyl), 2) PCN (Phosphate + CH_2_CH_2_N), 3) CG (Carbonyl + glycerol) groups, 4) CH_2_, and 5) CH_3_ methyl groups at the bilayer center.

**Figure 2:**
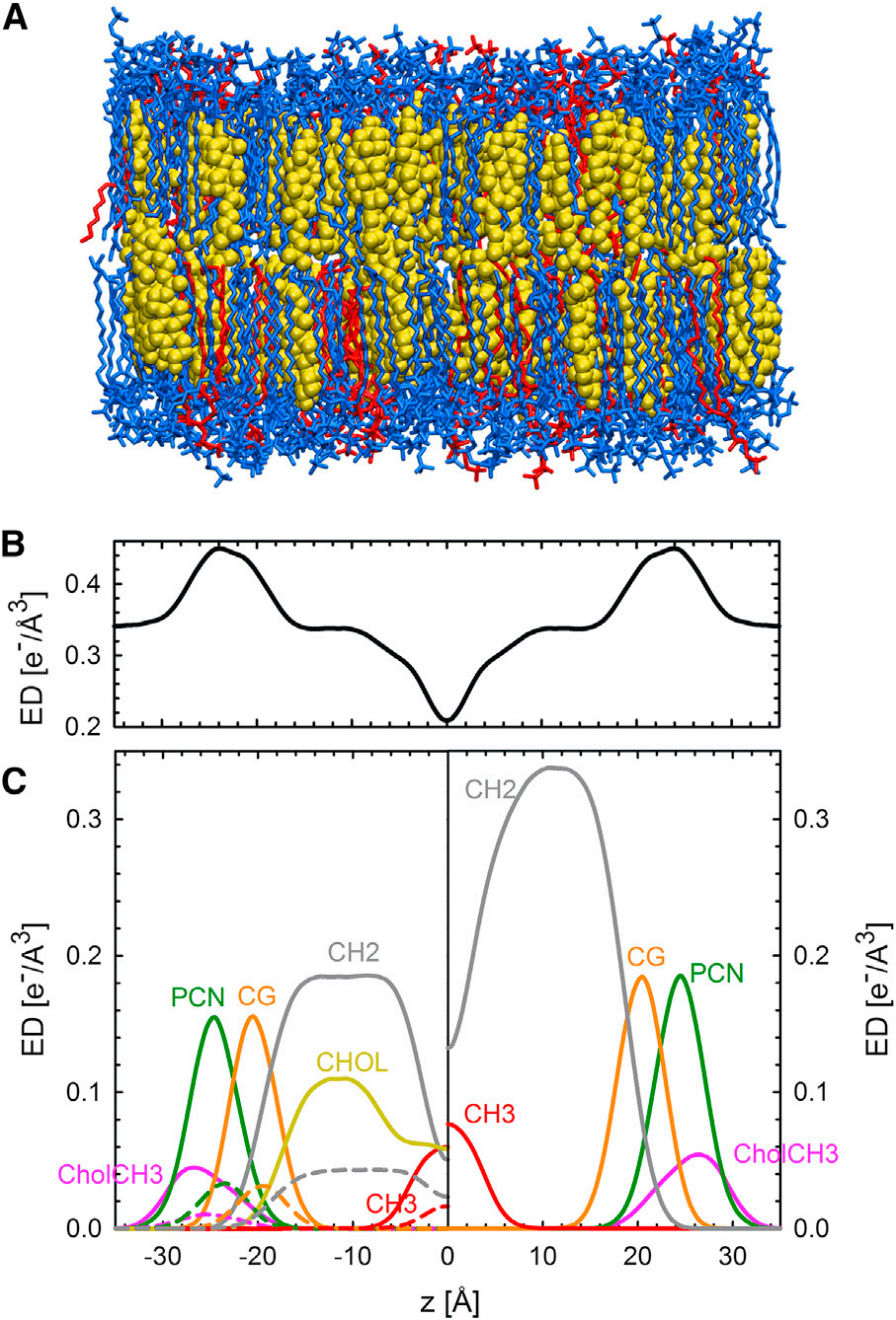
Parsing scheme of ternary lipid mixtures based on MD simulations of an *L*_*o*_ phase of DOPC/DPPC/CHOL (31). (*A*) Snapshot of the equilibrated system. DPPC lipids, *blue*; DOPC, *red*; cholesterol, *yellow*. (*B*) Calculated electron density profile. (*C*) Electron densities of molecular groups, calculated using SIMTOEXP (70). (*Left*) Individual contributions of DPPC (*solid lines*) and DOPC (*dashed lines*) for the CholCH_3_, PCN, CG, CH_2_, and CH_3_ groups. (*Yellow line*) Contribution of cholesterol. (*Right*) Condensed parsing scheme after merging individual contributions as detailed in the main text. To see this figure in color, go online.

This approach is further justified by its compatibility to previously reported molecular dynamics (MD) data (31), as demonstrated in Fig. 2. For details of the functional forms used to describe the individual groups, we refer to the literature (40,41,48). As detailed previously (41), a genetic algorithm was applied for fitting the global model to experimental data.

Membrane structural parameters such as hydrocarbon chain length *d*_*C*_, Luzzati thickness *d*_*B*_, water layer thickness *d*_*W*_, and the area per lipid *A* = 2*V*_*L*_/*d*_*B*_ were defined and calculated from the SDP profiles as described in Heftberger et al. (41). *V*_*L*_ is the total lipid volume, which is assumed to be given by the molecular-weighted average

(3) $$V_{L}\left(T\right)=\sum_{i}x_{i}\left(T\right)V_{i}\left(T\right),$$

where *x*_*i*_ values are the lipid molar ratios in *L*_*d*_ and *L*_*o*_ taken from Uppamoochikkal et al. (49) and Heberle et al. (50), *V*_*i*_ values are the corresponding molecular lipid volumes, and *T* is the temperature. Temperature-dependent *V*_*i*_ values were calculated according to the method of Koenig and Gawrisch (51), and the volume of cholesterol within lipid bilayers was taken to be 630 Å^3^ (52). Calculated tieline endpoint *V*_*L*_ were in good agreement (<2%) with experimental values determined by dilatometry (Supporting Material). Note that dilatometry yields a globally averaged value for the lipid volume and is thus not able to discern between *L*_*o*_ and *L*_*d*_ in the phase coexistence regime. Thus experimental *V*_*L*_ can be obtained for tieline endpoints, only. The temperature dependence of *x*_*i*_ was estimated by the lever rule using the experimentally determined *L*_*d*_ and *L*_*o*_ fractions (*c*_*Ld*_ and 1 − *c*_*Ld*_), assuming 1) that the inclination of the tieline remains constant, and 2) that the tieline length changes according to the *L*_*o*_ fraction with temperature (Supporting Material).

## Results and Discussion

### Establishing the global analysis for two phases

Our strategy to validate this analysis was as follows. 1) We evaluated tieline endpoint SAXS data. At endpoints, either *L*_*o*_ or *L*_*d*_ should exist as a single phase, thus allowing application of our previous analysis for homogeneous MLVs (41). 2) The phase coexistence model was applied to a composition close to the tieline midpoint and the achieved results were compared to endpoint data.

Phase boundaries, in particular, are subject to considerable uncertainties (8,49,53–55), whereas recent tieline orientation data are considered to be more reliable. In this study, we applied previously published compositional phase diagrams from Heberle et al. (50) and Uppamoochikkal et al. (49). Note that Uppamoochikkal et al. (49) used the phase diagram reported by Veatch et al. (54), and constructed tielines using x-ray scattering on oriented lipid films.

Our measurement strategy allowed for an independent check of these data, as follows: 1) Bragg peaks of all samples measured per tieline are required to overlap, if tieline orientation is correct (49); and 2) only a single lamellar lattice should be observed at the phase boundaries.

For all samples, including replicas, *L*_*o*_/*L*_*d*_ peak positions for tieline midpoints and endpoints matched, reassuring not only tieline orientation data, but also our sample preparation. The *L*_*d*_ endpoints of DOPC/DPPC/CHOL contained significant residual scattering from an *L*_*o*_ phase, revealing errors in the reported phase boundary. However, because *L*_*o*_ peaks overlapped with those of the *L*_*o*_ endpoint, we were able to subtract the *L*_*o*_ contribution (Fig. S1 in the Supporting Material). In independent experiments, using a laboratory x-ray camera, we determined for *B*_*t*2_ a new *L*_*d*_ endpoint by measuring several samples along the tieline until the *L*_*o*_ contributions vanished. The new endpoint composition is 0.748/0.124/0.128 (Fig. 1); corresponding SAXS data are shown in Fig. S2.

**Figure 3:**
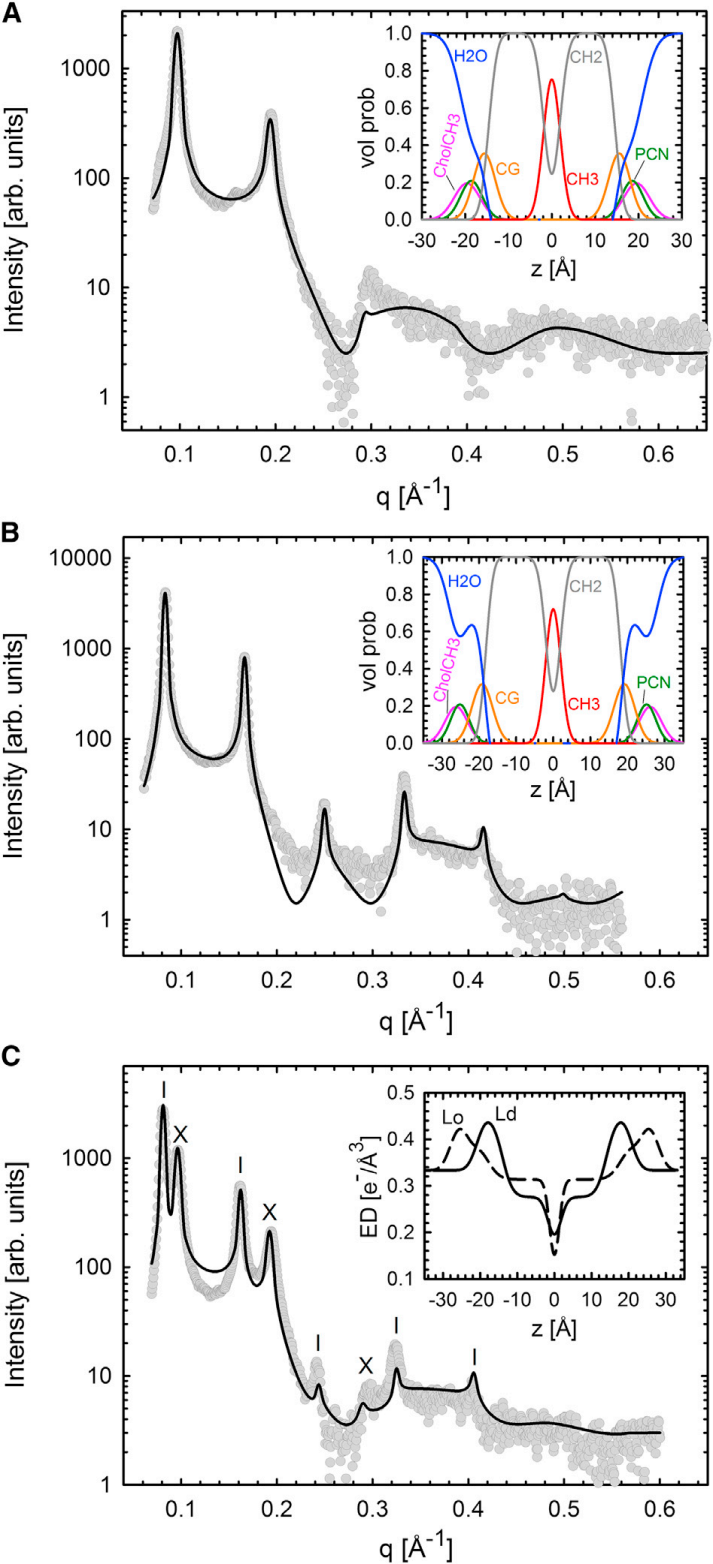
Validation of the global analysis for two coexisting phases for the *A*_*t*1_ tieline (*T* = 22°C). (*A* and *B*) Fits to *L*_*d*_ and *L*_*o*_ endpoint data, respectively. (*Insets*, both panels) Derived volume probability distributions. (*C*) Best fit to SAXS data at the *A*_*t*1_ tieline midpoint. Bragg reflections of *L*_*o*_ (*dashes*) and *L*_*d*_ (*crosses*) domains. (*Inset*) ED profiles for *L*_*o*_ and *L*_*d*_ phases. To see this figure in color, go online.

Fig. 3 details the results and analysis of the *A*_*t*1_ tieline. The *L*_*d*_ endpoint in this study showed some residual *L*_*o*_ contribution. However, it was small enough to be neglected. Global fits to tieline endpoints and midpoints show that our model is able to capture both the single-phase and two-phase coexistence, respectively. Insets to Fig. 3 show the volume probability distributions of individual quasi-molecular groups (see previous section) for *L*_*o*_, *L*_*d*_ endpoints and the resulting electron density (ED) profiles for the coexisting case. The absolute ED in the hydrocarbon chain region of the *L*_*o*_ phase is significantly higher than in *L*_*d*_. This can be explained by the higher amount of cholesterol in the *L*_*o*_ phase, with substantial ED contributions from the sterol ring.

All structural parameters for coexisting domains agreed remarkably to tieline endpoints (Tables 1 and S2), thereby validating our analysis. For example, the membrane thickness *d*_*B*_ for *L*_*d*_ and *L*_*o*_ endpoints is 37.5 and 49.7 Å, respectively. This compares well to *d*_*B*_ = 38.1 and 48.6 Å for *L*_*d*_ and *L*_*o*_ in the coexistence regime, meaning: differences are within <1 Å. Note that height differences Δ*d*_*B*_ between *L*_*d*_ and *L*_*o*_ domains are in the same range as those reported between detergent-resistant and detergent-soluble membranes (22).

The two-phase analysis was further tested by checking whether a decrease of the hydrocarbon chain length of the mixture’s high-melting lipid leads to reasonable changes in domain structure. Exchanging DSPC with DPPC affected mainly the structure of the *L*_*o*_ phase. Picking for example the *t*1 tielines, *d*_*B*_ decreased by 2.6 Å, whereas only a minor decrease of 0.6 Å was found for *L*_*d*_. Similar changes were found for other tielines, including *d*_*C*_-values (Table 1). Our findings are in excellent agreement with tieline orientation (Fig. 1); because the high-melting lipid is located in *L*_*o*_ domains, we observe a thinning by exchanging DSPC to DPPC, which contains two CH_2_ groups less per acyl chain. Kučerka et al. (47) reported a similar thickness difference for pure DSPC and DPPC bilayers. *L*_*d*_ phases contain mainly DOPC and are consequently barely effected by the lipid exchange. Further structural parameters for *L*_*d*_ (Table 1) are close to that of pure DOPC (40).

Finally, we compare areas per lipid, *A*, which differ significantly between *L*_*d*_ and *L*_*o*_ phases (Table 1). The *L*_*d*_ domains exhibited *A* values between 60 and 65 Å^2^, which is in the range of values reported for fluid single lipid bilayers (40,47). Areas are ∼20 Å^2^ smaller for *L*_*o*_ domains. The main reason for this difference is the condensing effect of cholesterol, which was previously reported for several binary phosphatidylcholine/cholesterol mixtures (48,56–58) and is now also observed for coexisting *L*_*o*_/*L*_*d*_ domains. Another manifestation of this ordering effect is the decrease of the bending fluctuation parameter from *η* ∼ 0.08 (*L*_*d*_) to ∼0.03 (*L*_*o*_).

Thus, concluding this section: our global SAXS data analysis yields, within typical uncertainties of the SDP model, robust high-resolution results for structure and fluctuations of coexisting *L*_*o*_/*L*_*d*_ domains. This allows us to obtain reliable insights on changes of these parameters, e.g., as a function of composition or temperature. Results of such experiments are presented in the following sections.

### Effect of cholesterol on domains

To study the influence of raising cholesterol concentration, we compare the *t*1 and *t*2 tieline midpoints for both ternary mixtures. Scattering profiles and fits are plotted in Fig. 3 (Figs. S3, S4, and S5), while results for structural and elastic parameters are presented in Table 1. For both systems studied, *d* increased by ∼0.75 Å for *L*_*d*_ domains, but decreased by ∼2 Å for *L*_*o*_ upon increasing cholesterol content. Our analysis revealed that the increase of *d* for *L*_*d*_ is mainly due to a thickening of its bilayer, whereas only approximately one-third of the decrease of *d* for *L*_*o*_ can be attributed to *d*_*B*_. A decrease of *d*_*B*_ for *L*_*o*_ upon increasing cholesterol concentration may seem counterintuitive, but the marginal additional ordering effect due to more cholesterol is overcompensated by a reduction of the high-melting lipid concentration. Most of the change in *d* for *L*_*o*_ is due to a decrease of the interstitial water layer (1–2 Å), which may originate either from an increase in net attractive forces, or a decrease in net repulsive forces between *L*_*o*_ domains. This effect cannot be attributed to an increased bending rigidity due to the higher cholesterol content (59), because the fluctuations did not decrease (Table 1). Instead, a decrease of hydration or an increase of van der Waals forces might be the reason.

The area per lipid was found to be smaller for *L*_*d*_ domains of the *t*2 tielines (Table 1), which can be attributed to the well-known condensing effect of cholesterol (58). For *L*_*o*_ domains, changes for *A* were found to be insignificant (within experimental uncertainty). However, it is interesting to note that the variation of *A* for *L*_*o*_ even across DOPC/DPPC/CHOL and DOPC/DSPC/CHOL is within <±2%. This indicates that the average value *A* ∼ 43.6 Å could be the tightest possible packing of lipids in the *L*_*o*_ phase. More structural data on *L*_*o*_ would certainly be needed to validate this notion.

### Temperature dependence of *L*_*o*_/*L*_*d*_ domains

**Figure 4:**
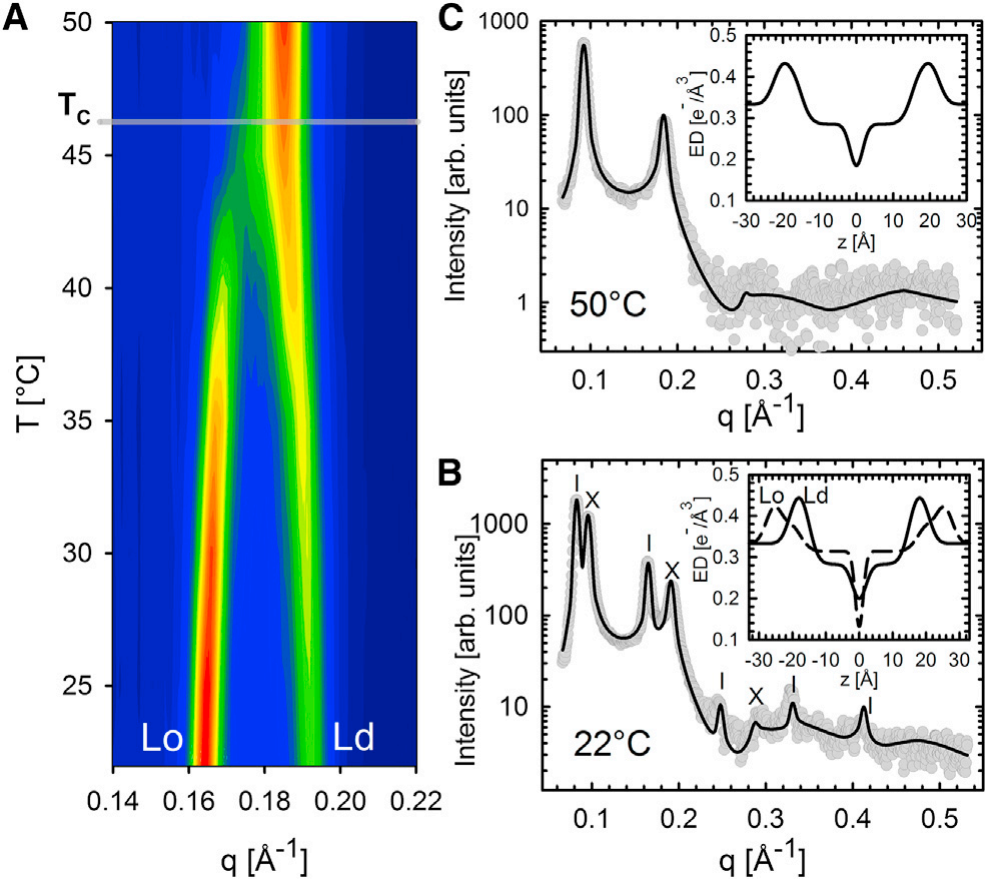
Temperature behavior of DOPC/DSPC/CHOL (tieline *t*2) as revealed by SAXS. (*A*) Contour plot of the second-order Bragg reflections indicated as *L*_*o*_ and *L*_*d*_. Note that the smooth appearance of data is due to an interpolation procedure between the individual frames. The critical temperature *T*_*C*_ is between 45 and 50°C. At >*T*_*C*_, only a single lamellar lattice is observed. (*B*) Measured scattering at 22°C with the indicated Bragg reflections for *L*_*o*_ (*dashes*) and *L*_*d*_ (*crosses*) domains. (*C*) The same, for 50°C. (*Solid lines*) Best fits. (*Insets*, both panels) Resulting ED profiles for *L*_*o*_ and *L*_*d*_ phases. To see this figure in color, go online.

**Figure 5:**
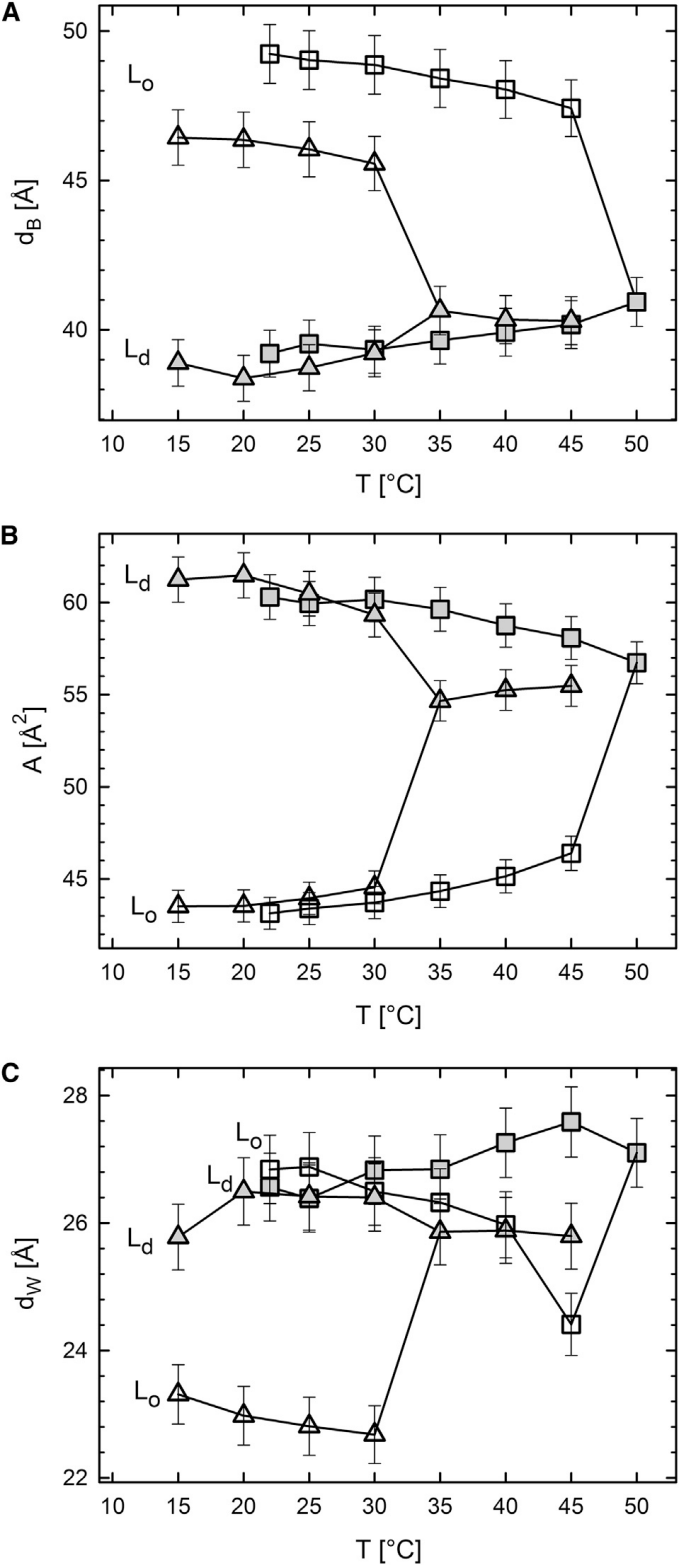
Temperature dependence of structural parameters of DOPC/DSPC/CHOL (*squares*) and DOPC/DPPC/CHOL (*triangles*), starting from the *t*1 tieline midpoints (Fig. 1). (*A*) Bilayer thickness, (*B*) area per lipid, and (*C*) water layer thickness, for *L*_*d*_ (*solid symbols*) and *L*_*o*_ (*open symbols*) domains as a function of temperature.

Starting from the reported compositional phase diagrams (Fig. 1), we increased temperature in steps of 5°C until we reached a homogeneous phase. The transition is observed as a merging of the *L*_*o*_ and *L*_*d*_ lattices into a single *L*_*d*_ phase lattice (Fig. 4). For DOPC/DSPC/CHOL, the transition at *T*_*C*_ occurred between 45 and 50°C, and for DOPC/DPPC/CHOL, between 30 and 35°C. Note that our temperature resolution does not allow us to determine *T*_*C*_ with high accuracy. Fig. 5 *A* compares the results for *d*_*B*_ of *A*_*t*1_ and *B*_*t*1_. The *L*_*o*_ phase of *B*_*t*1_ was found to be 3 Å thinner than that of *A*_*t*1_. Because changes with temperature are similar for both *t*1 and *t*2 tielines (Figs. 5 and S6), we can therefore limit the discussion to the *t*1 tielines. In the temperature range of 22–45°C *d*_*B*_ of *L*_*d*_ domains increased monotonously by ∼1–1.5 Å, whereas *d*_*B*_ for *L*_*o*_ decreased at the same time by 2 Å. Close to *T*_*C*_, these changes are significantly accelerated. Above the transition temperature, *d*_*B*_ is similar to that of the *L*_*d*_ phase just below *T*_*C*_. Interestingly, *d*_*B*_ is approximately equal for DSPC- and DPPC-containing samples above *T*_*C*_, including the *t*2 tielines (Fig. S6) despite the difference in hydrocarbon chain length.

The thickness of single-phase fluid lipid bilayers typically decreases with increasing temperature (47,60,61). Thus, the thickening of the *L*_*d*_ domains upon approaching *T*_*C*_ from below is surprising. Davis and Schmidt (23) recently suggested, based on NMR data, that the cholesterol fraction in *L*_*o*_ decreases with temperature. Consequently, *L*_*d*_ would get enriched in cholesterol. Because of the associated condensation effect of cholesterol, one would then expect an increase of the *L*_*d*_ domain thickness. Our results consequently corroborate this scenario.

**Figure 6:**
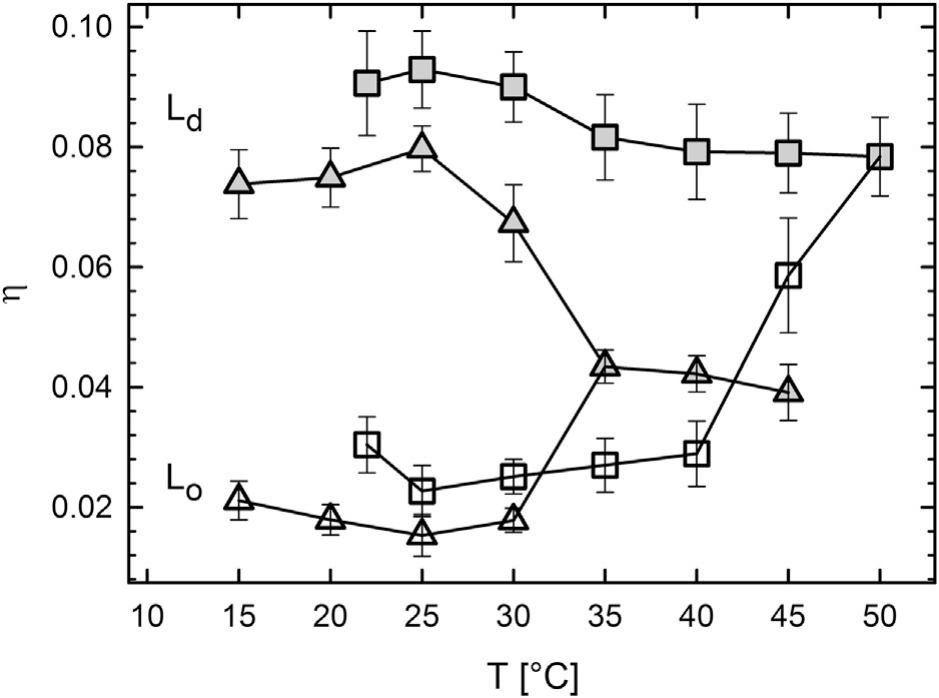
Temperature dependence of bending fluctuations of coexisting DOPC/DSPC/CHOL (*squares*) and DOPC/DPPC/CHOL (*triangles*) domains, for *t*1 tieline compositions (Fig. 1 and Table S1). (*Shaded symbols*) Results for *L*_*d*_ domain; (*open symbols*) results for *L*_*o*_ domain.

The area per lipid is inversely proportional to *d*_*B*_. Hence the temperature changes of *A* are similar to *d*_*B*_, but just with inverted trends, i.e., *A* decreases for *L*_*d*_ and increases for *L*_*o*_, as observed in Fig. 5 *B*. We further note that areas above *T*_*C*_ are alike for all systems and tielines studied, which appears reasonable in view of the similar *A*-values reported for single-component DPPC and DSPC membranes (47). Changes of the water layers in turn appear to be decoupled from the trends of *d*_*B*_ and *A*. We found a general increase of *d*_*W*_ for *L*_*d*_ domains and a decrease for *L*_*o*_ domains (Fig. 5 *C*) below *T*_*C*_, with changes close to *T*_*C*_ being more pronounced for the *L*_*o*_ phase. These findings are not straightforward to explain, in particular because the Caillé parameter did not show a strong increase of bending fluctuations for *L*_*d*_, or decrease for *L*_*o*_, respectively. Instead, an overall decrease in *η* was found for *L*_*d*_ (Fig. 6) and an increase in the vicinity of *T*_*C*_ for *L*_*o*_. These two trends can be explained by the temperature-driven diffusion of cholesterol to *L*_*d*_, as discussed above. Specific changes in *d*_*W*_ in turn appear to be caused by other influences on intermembrane interactions. Additional experiments, such as a combination of SAXS with osmotic stress (62), are needed to address this issue properly.

**Figure 7:**
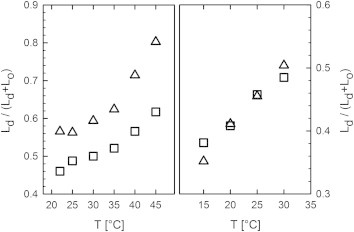
Variation of *L*_*d*_ phase fraction with temperature. (*Left*) Results for DOPC/DSPC/CHOL. (*Right*) Results for DOPC/DPPC/CHOL. (*Squares*) Results for *t*1 tielines; (*triangles*) results for *t*2 tielines.

Melting of *L*_*o*_ domains can be further assessed by *c*_*Ld*_, corresponding to the *L*_*d*_ phase fraction (Eq. 2). All studied systems show a steady increase of *c*_*Ld*_ as *T*_*C*_ is approached from below, while the *t*2 composition of DOPC/DSPC/CHOL exhibited the largest overall *L*_*d*_ fraction (Fig. 7). The increase of *c*_*Ld*_ also signifies that the *L*_*d*_ tieline endpoints approach the chosen midpoints more rapidly than the *L*_*o*_ endpoints. Hence, the *L*_*o*_/*L*_*d*_ coexistence regime reduces asymmetrically with temperature, i.e., closes-in faster on the *L*_*d*_ boundary than on the *L*_*o*_ boundary, in agreement with Buboltz et al. (63) (and see the Supporting Material).

Finally, it is interesting to compare the relative increase of the *L*_*d*_ fraction in the studied temperature range. For DOPC/DSPC/CHOL, *c*_*Ld*_ increased with temperature by 16% for the *t*1 and 25% for the *t*2 tieline, respectively. Differences are smaller for DOPC/DPPC/CHOL, with Δ*c*_*Ld*_ = 9% for *t*1 and 16% for *t*2, but here changes are more pronounced at higher cholesterol content. Hence, increasing cholesterol concentration appears to promote melting of *L*_*o*_. This can be understood by reviewing the height differences between *L*_*d*_ and *L*_*o*_ domains (Table 1). For DSPC-containing mixtures, Δ*d*_*B*_ =11.3 Å for the *t*1 tieline and Δ*d*_*B*_ =10 Å or *t*2, whereas Δ*d*_*B*_ = 9.7 Å and Δ*d*_*B*_ = 7.5 Å for the *t*1 and *t*2 tielines in DOPC/DPPC/CHOL. Thus, Δ*d*_*B*_ decreases with cholesterol concentration for both systems. The height differences are related to the line tension *γ* between *L*_*d*_ and *L*_*o*_ domains. In particular, Akimov et al. (30) showed that *γ* ∝ $\Delta d_{B}^{2}$. Consequently, the cholesterol-induced decrease of Δ*d*_*B*_ leads to a lowering of *γ*, facilitating the melting of *L*_*o*_ domains.

## Conclusions

We introduced a global SAXS data analysis technique, which yields structural and elastic properties of coexisting *L*_*o*_/*L*_*d*_ domains in multilamellar vesicles. The model captures 1) high structural resolution by incorporating the SDP model (40) and 2) bending fluctuations through a Bragg peak line-shape analysis in terms of the Caillé theory structure factor (39). The method has been verified on DOPC/DSPC/CHOL and DOPC/DPPC/CHOL mixtures by comparing tieline endpoint with midpoint data of corresponding phase coexistence samples, and by essaying whether it captures the effects of chain length increase for *L*_*o*_ domains, such as thickness increase or a decrease of lipid area.

We further characterized two tielines for each ternary mixture to study effects of increased cholesterol concentration. Interestingly, additional cholesterol affected mostly structural properties of the *L*_*d*_ phase (increase of *d*_*B*_ and *d*_*C*_, decrease of *A*, and decrease of *η*), whereas *L*_*o*_ appeared to be already saturated. A further effect of higher cholesterol concentration was a decrease of the thickness difference between *L*_*o*_ and *L*_*d*_ domains, leading to a lowering of line tension and consequently to a destabilization of *L*_*o*_ domains that is somehow analogous to the well-known order/disorder effect of cholesterol in binary lipid mixtures (64). The temperature behavior revealed structural and elastic changes during melting of *L*_*o*_ domains, which suggest that cholesterol diffuses into *L*_*d*_ domains even below *T*_*C*_.

Because of its ability to analyze phase coexistence data without using labels, our technique should be able contribute to resolving several open questions in the field. One of the many controversial issues of raftlike lipid mixtures, for example, is their critical behavior across the transition into a homogeneous phase. According to theory, any defined order parameter should vary ∝(*T*_*C*_ – *T*)^*β*^, where the critical exponent *β* is either 0.125 or 0.325, depending on whether the system follows the two- or three-dimensional Ising model, respectively (23,65–68). Fluorescence microscopy experiments on compositional fluctuations in the vicinity of *T*_*C*_ revealed a two-dimensional Ising model-like behavior (65–67). In contrast, atomic force measurements on the height-difference of *L*_*o*_/*L*_*d*_ (68) and first momenta of NMR spectra (23) reported critical exponents favoring the three-dimensional Ising model. We analyzed the height difference between *L*_*o*_ and *L*_*d*_ phases across *T*_*C*_ to determine a critical exponent (Fig. S8). Our results favor the two-dimensional Ising model, but the apparently coarse temperature steps preclude us from any firm statement. Future studies will be designed to exactly address this issue. Another interesting application for our technique will be to predict protein activity and partitioning in domains (17), which can be achieved by adding information on spontaneous curvatures (69) and bending elasticities (31,33) of *L*_*o*_ and *L*_*d*_ domains. This work is underway in our laboratory.

## Figures and Tables

**Table 1:** Structural results and bending fluctuations for coexisting *L*_d_/*L*_o_ domains

Term	*d*_*B*_	*A*	*d*_*W*_	*d*_*C*_	*η*
*A*_*t*1_(*L*_*d*_)	38.5	63.1	26.6	14.5	0.091
*A*_*t*1_(*L*_*o*_)	49.8	43.2	27.6	18.8	0.030
*A*_*t*2_(*L*_*d*_)	39.2	60.3	26.5	14.9	0.092
*A*_*t*2_(*L*_*o*_)	49.2	43.1	26.8	18.5	0.029
*B*_*t*1_(*L*_*d*_)	37.9	64.9	26.0	14.2	0.074
*B*_*t*1_(*L*_*o*_)	47.2	44.4	25.1	17.7	0.021
*B*_*t*2_(*L*_*d*_)	38.9	61.2	25.8	14.7	0.068
*B*_*t*2_(*L*_*o*_)	46.4	43.5	23.3	17.3	0.024

Parameter uncertainties are <2%.

## Supporting Material

Document S1. Supporting Materials and Methods, nine figures, and two tables

Document S2. Article plus Supporting Material
