# Supplementary material for: In Situ Determination of Structure and Fluctuations of Coexisting Fluid Membrane Domains
Source: Biophys J. 2015 Feb 17;108(4):854–62. doi: 10.1016/j.bpj.2014.11.3488 (PMC4336378; doi:10.1016/j.bpj.2014.11.3488)
Supplement: Document S1. Supporting Materials and Methods, nine figures, and two tables [file mmc1.pdf]

Supporting Material

In situ determination of structure and fluctuations of coexisting fluid membrane domains

Peter Heftberger,[†,‡] Benjamin Kollmitzer,[†,‡] Alexander A. Rieder,[†,‡] Heinz Amenitsch[§], and Georg Pabst[†,‡] *

[†] *University of Graz, Institute of Molecular Biosciences, Biophysics Division, NAWI Graz, Humboldtstr. 50/III, A-8010 Graz, Austria.*
[‡] *BioTechMed-Graz, Austria.*
[§] *Graz University of Technology, Institute of Inorganic Chemistry, Stremayrgasse 9/IV, A-8010 Graz, Austria.*

[*] Correspondence: georg.pabst@uni-graz.at

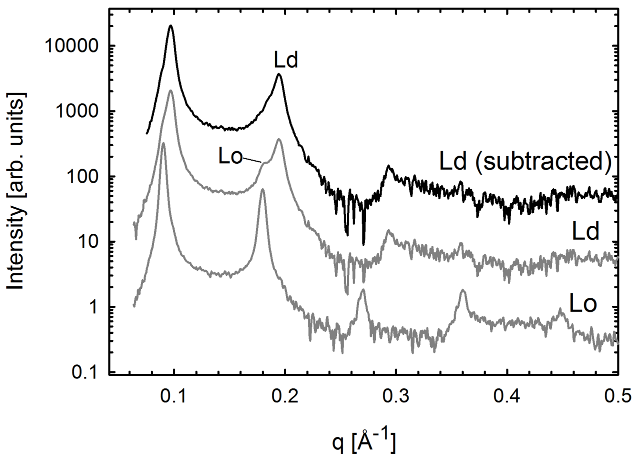

Figure S1: Subtraction of L_o contribution from L_d endpoint of B_{t2}. Scattering intensities are scaled by a constant for clarity. Second order Bragg reflections of L_o and L_d phases are indicated. Because of the overlap of L_o peaks for L_o and L_d endpoints (gray lines), we were able to subtract a fraction of L_o from the L_d endpoint sample. The result is shown in the top scattering pattern (black line).

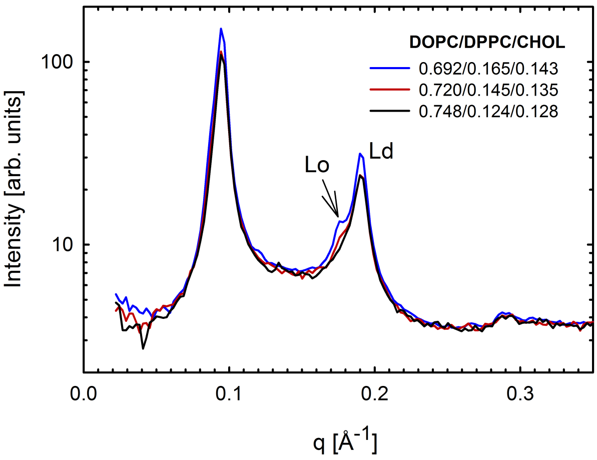

Figure S2: Determination of new L_d tieline endpoint for B_{t2}. Scattering patterns for three different lipid compositions, extending the B_{t2} tieline (1) toward the pure L_d regime (same angle), are shown. Data were recorded on a S3-Micro compact Kratky camera (Hecus X-ray Systems, Graz, Austria) at 15 °C. The second order Bragg peak clearly indicates L_o contamination. No residual L_o was observed for the DOPC/DPPC/CHOL composition 0.748/0.124/0.128 (molar fractions).

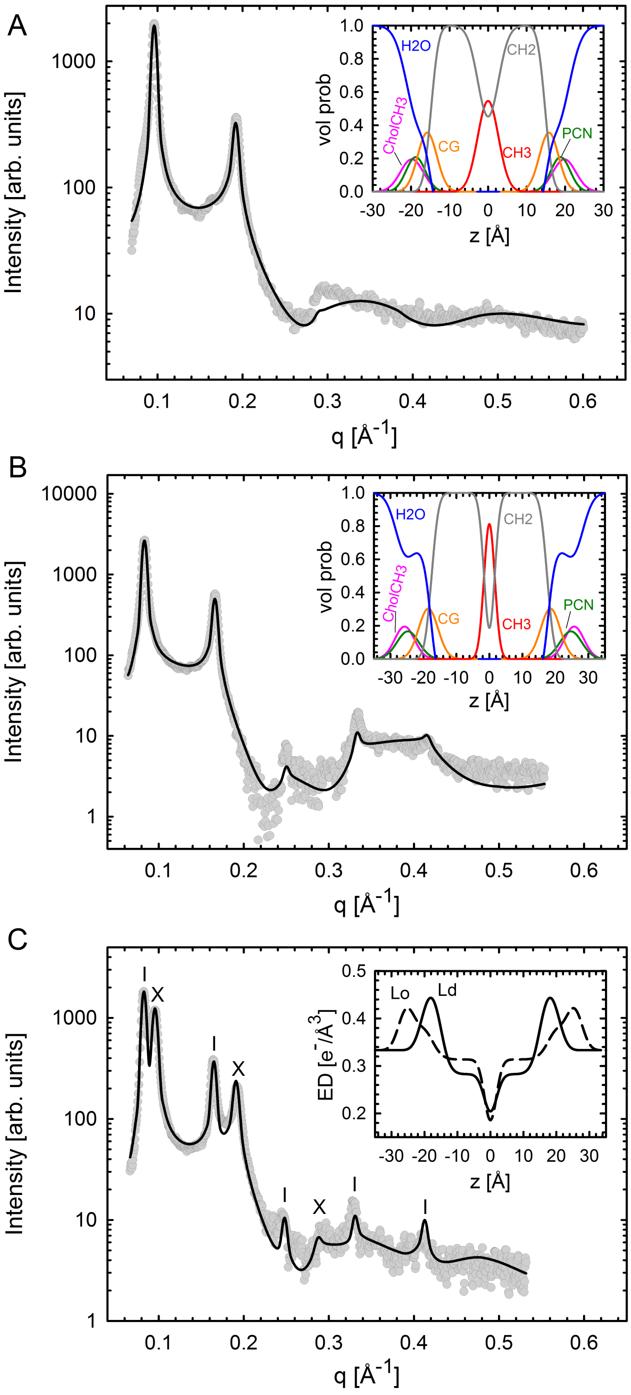

Figure S3: Validation of the global analysis for two coexisting phases for the A_{t2} tieline ($T =$ 22 °C). Panels A and B show fits to L_d and L_o endpoint data, respectively. The insets to both panels give the derived volume probability distributions. Panel C shows the best fit to SAXS data at the A_{t2} tieline midpoint. Bragg reflections of L_o and L_d domains are indicated with dashes and crosses, respectively. The corresponding inset gives the ED profiles for L_o and L_d phases.

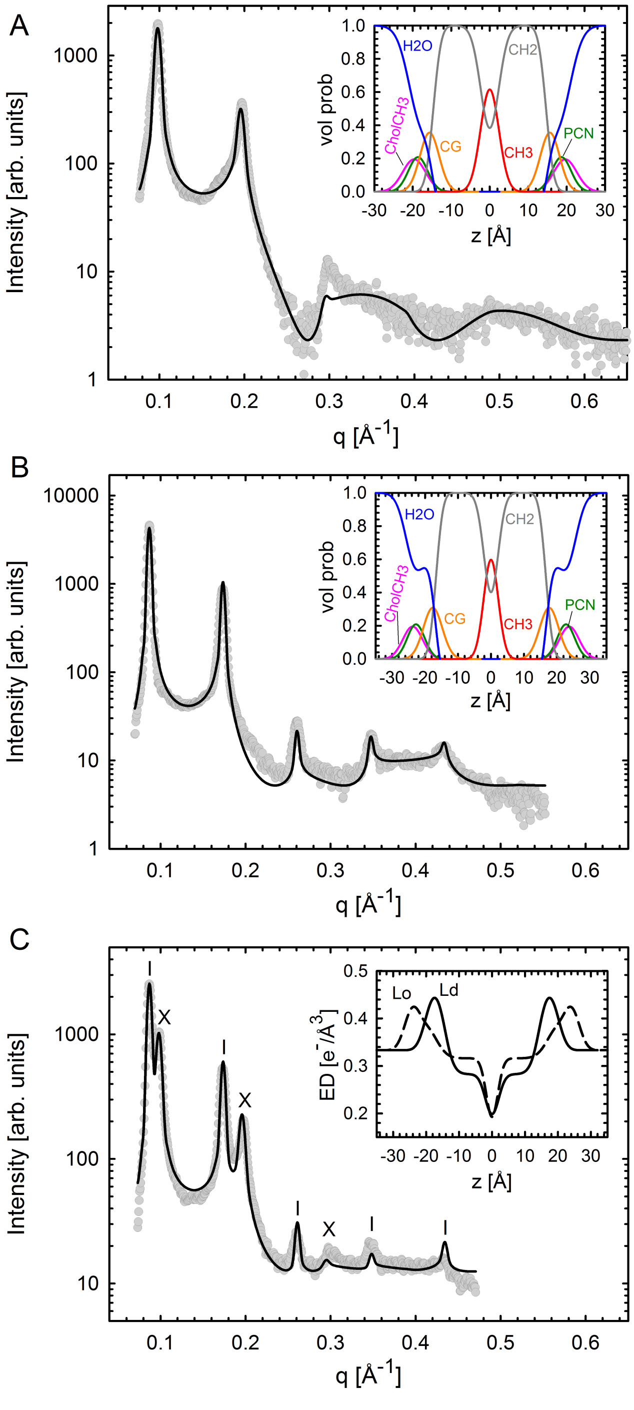

Figure S4: Validation of the global analysis for two coexisting phases for the B_{t1} tieline ($T = 15\,^\circ$C). Panels A and B show fits to L_d and L_o endpoint data, respectively. The insets to both panels give the derived volume probability distributions. Panel C shows the best fit to SAXS data at the B_{t1} tieline midpoint. Bragg reflections of L_o and L_d domains are indicated with dashes and crosses, respectively. The corresponding inset gives the ED profiles for L_o and L_d phases.

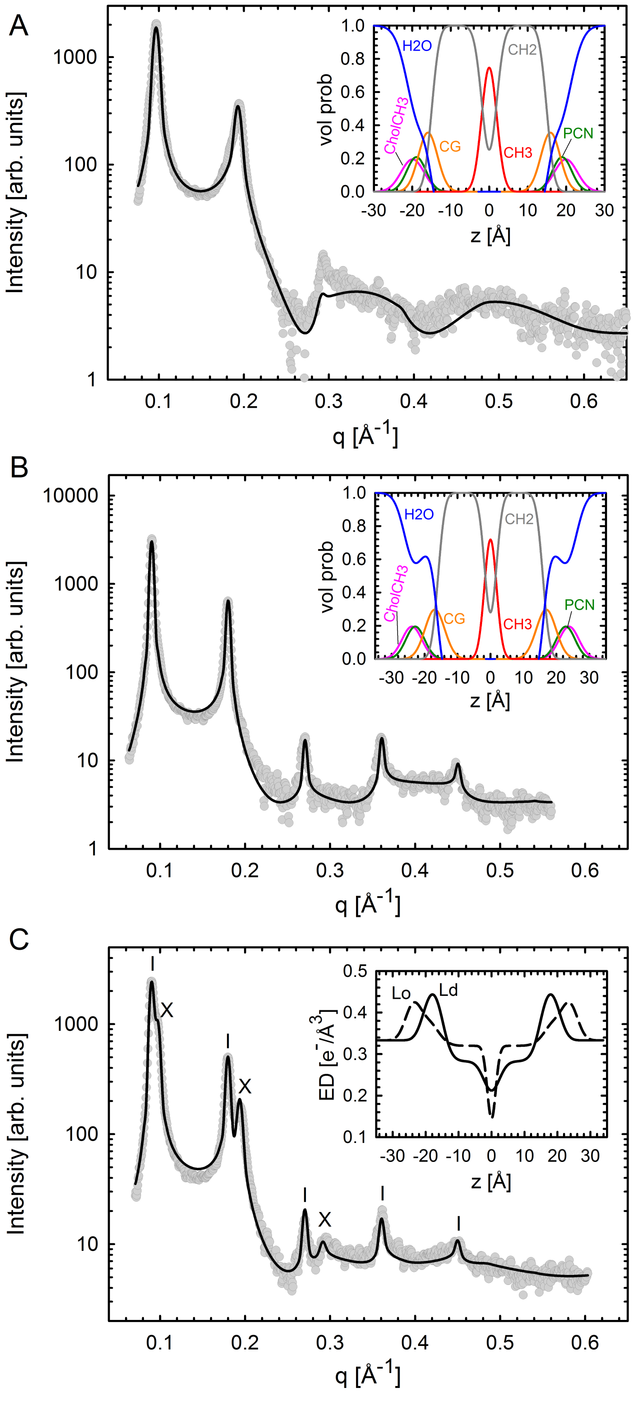

Figure S5: Validation of the global analysis for two coexisting phases for the B_{t2} tieline ($T = 15\,^\circ$C). Panels A and B show fits to L_d and L_o endpoint data, respectively. The insets to both panels give the derived volume probability distributions. Panel C shows the best fit to SAXS data at the B_{t2} tieline midpoint. Bragg reflections of L_o and L_d domains are indicated with dashes and crosses, respectively. The corresponding inset gives the ED profiles for L_o and L_d phases.

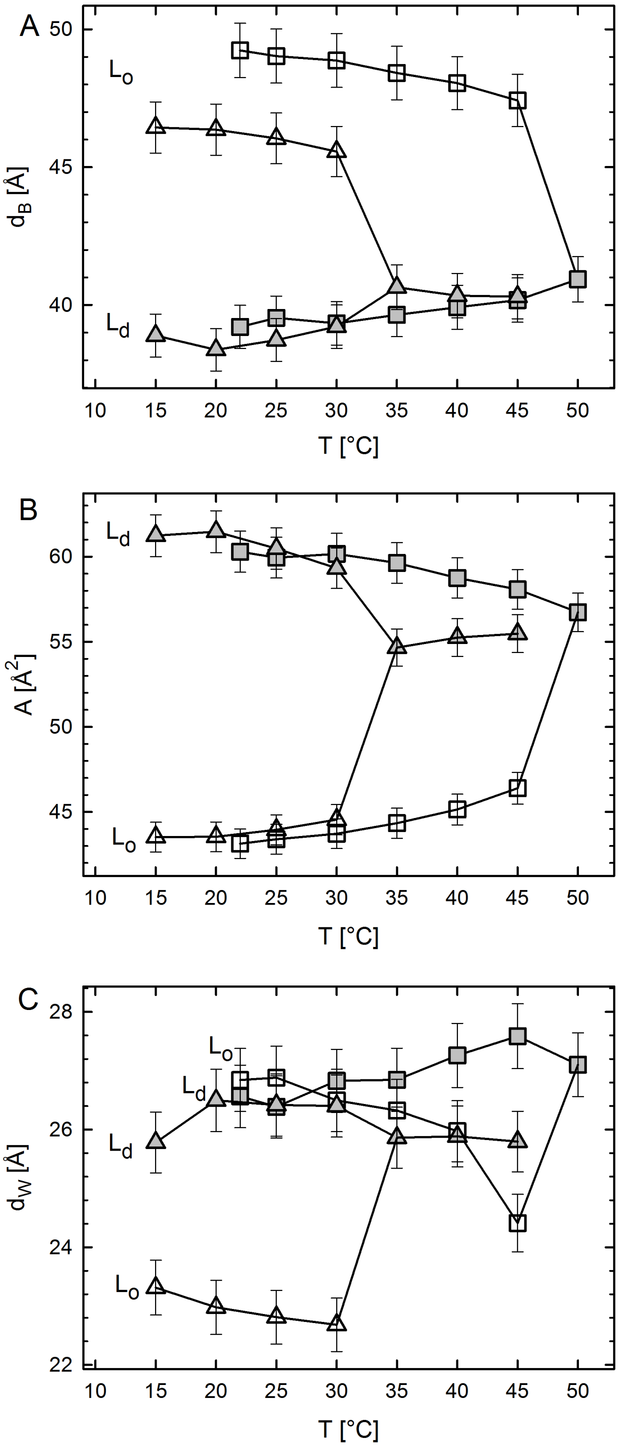

Figure S6: Temperature dependence of structural parameters of coexisting DOPC/DSPC/CHOL (squares) and DOPC/DPPC/CHOL (triangles) domains for $t2$ tieline compositions (Fig. 1, Table S1). Panels A–C show the bilayer thickness, area per lipid, and water layer thickness, respectively for L_d (solid symbols) and L_o (open symbols) domains.

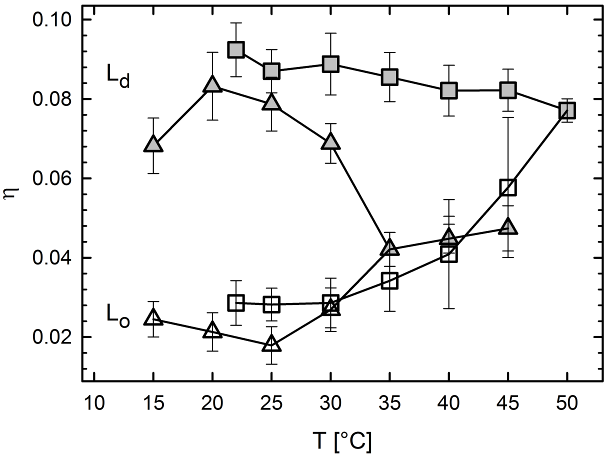

Figure S7: Temperature dependence of bending fluctuations of coexisting DOPC/DSPC/CHOL (squares) and DOPC/DPPC/CHOL (triangles) domains for $t2$ tieline compositions (Fig. 1, Table S1). Solid symbols show results for L_d and open symbols for L_o domains, respectively.

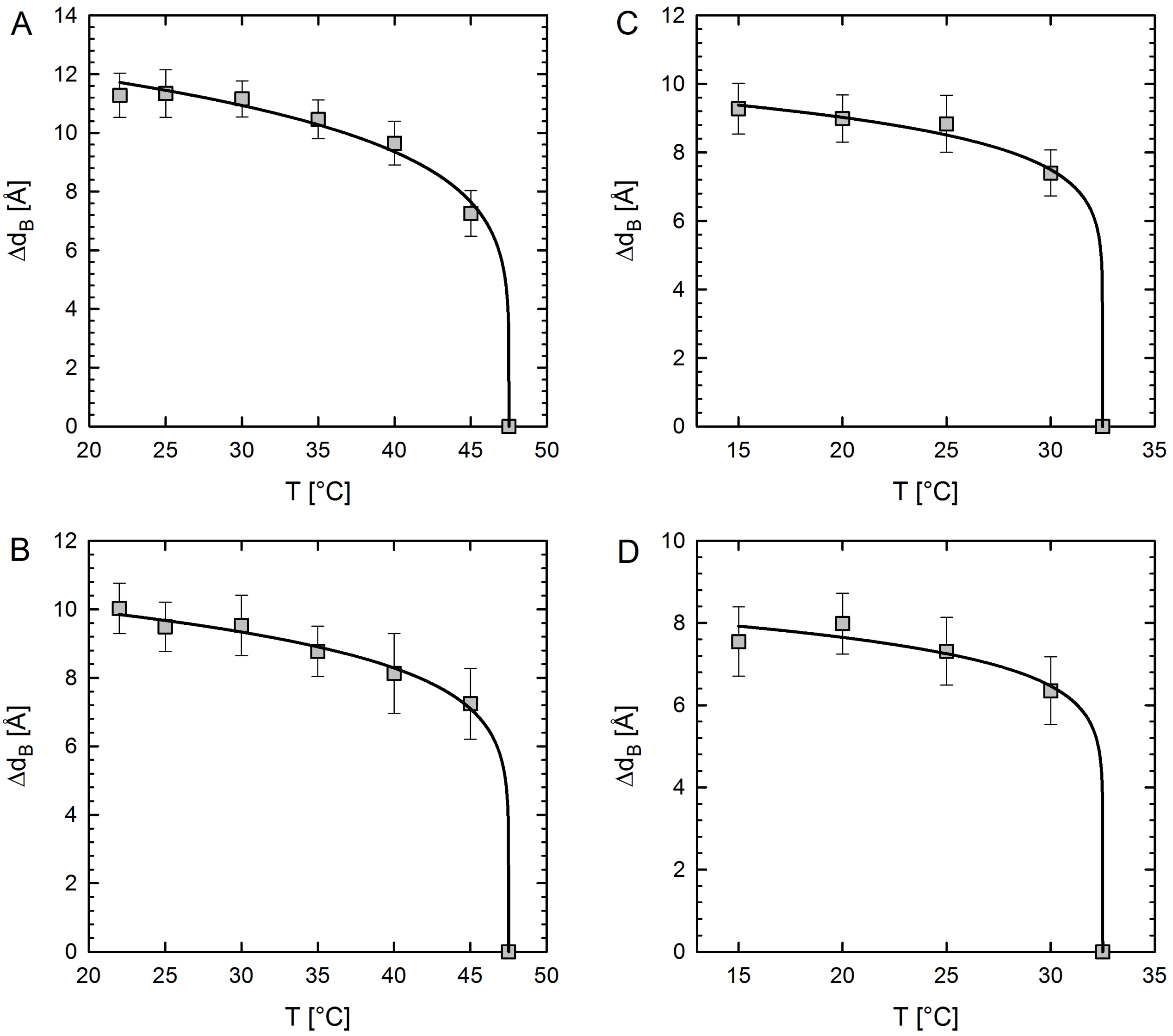

Figure S8: Critical behavior of height difference Δd_B between L_o and L_d domains of tieline midpoint samples A_{t1} (A), A_{t2} (B), B_{t1} (C) and B_{t2} (D). Solid lines correspond to the best fits using $\Delta d_B \propto (T_C - T)^{\beta}$. T_C was estimated to be 47.5 °C for (A,B) and 32.5 °C for (C,D) yielding the critical exponents $\beta_A = 0.18$, $\beta_B = 0.14$, $\beta_C = 0.115$ and $\beta_D = 0.105$. To evaluate the influence of our choice of T_C, T_C was varied between the extremes of the experimental temperature interval. For $T_C^{min} = 46/31$ °C (for A,B/C,D), we obtained $\beta_A = 0.14$, $\beta_B = 0.11$, $\beta_C = 0.08$ and $\beta_D = 0.08$. For $T_C^{max} = 50/35$ °C (for A,B/C,D), we found $\beta_A = 0.24$, $\beta_B = 0.19$, $\beta_C = 0.16$ and $\beta_D = 0.24$.

Table S1: Lipid compositions (molar fractions) of measured samples according to published compositional phase diagrams (1, 2). Two tielines with a lower (A_{t1}, B_{t1}) and higher (A_{t2}, B_{t2}) cholesterol concentration were studied (see Fig. 1).

		DOPC	DSPC	CHOL
	L_d	0.79	0.09	0.12
A_{t1}	L_o	0.05	0.65	0.30
	L_d/L_o	0.42	0.37	0.21
	L_d	0.74	0.09	0.17
A_{t2}	L_o	0.12	0.56	0.32
	L_d/L_o	0.46	0.30	0.24
		DOPC	DPPC	CHOL
	L_d	0.70	0.23	0.07
B_{t1}	L_o	0.11	0.68	0.21
	L_d/L_o	0.37	0.47	0.16
	L_d	0.66	0.19	0.15
B_{t2}	L_o	0.12	0.58	0.30
	L_d/L_o	0.36	0.41	0.23

Table S2: Structural results and bending fluctuations for the studied tieline endpoints. V_L are the calculated and V_L' the experimentally determined molecular volumes of an effective lipid molecule, including contributions from all three components (for details, see subsequent section). Parameter uncertainties are $< 2\,\%$.

	d_B[Å]	A[Å^2]	d_W[Å]	d_C[Å]	η	V_L[Å^3]	V_L'[Å^3]
A_{t1}-L_d	38.6	62.9	25.8	14.6	0.079	1215	1233
A_{t1}-L_o	48.6	44.2	26.9	18.2	0.047	1075	1063
A_{t2}-L_d	39.1	60.5	26.3	14.8	0.097	1183	1200
A_{t2}-L_d	48.0	44.3	27.4	17.9	0.060	1065	1058
B_{t1}-L_d	38.8	63.5	25.2	14.7	0.082	1231	1227
B_{t1}-L_o	46.6	44.9	25.8	17.4	0.027	1047	1049
B_{t2}-L_d	39.3	60.6	25.6	14.9	0.078	1191	1183
B_{t2}-L_o	45.9	44.0	23.7	17.0	0.028	1010	1015

Lipid volume calculation

The calculated lipid volumes of the ternary mixtures described in Material and Methods, were compared to experimentally determined volumes using the density sound analyzer DSA5000 (Anton Paar, Graz, Austria). Specifically, experiments yielded the partial specific volume (see e.g. (3))

$$v_s = \frac{1}{\rho_0}\left(1 - \frac{\rho_s - \rho_0}{c}\right), \tag{1}$$

where ρ_0 is the density of the solvent, ρ_s density of the solution and c the solute concentration. The experimental volume per molecule is then calculated according to Greenwood et al. as (4):

$$V_L' = \frac{v_s}{N_A}(x_1 M_1 + x_2 M_2 + x_3 M_3), \tag{2}$$

where N_A is Avogadro's number, x_i molar fraction and M_i the molecular weights.

Alternatively, volumes can be calculated by (5)

$$V_L(T) = V_H + n_{CH} V_{CH}(T) + n_{CH2} V_{CH2}(T) + n_{CH3} V_{CH3}(T), \tag{3}$$

where the head group volume V_H is kept fixed at 319 Å^3. V_{CH}, V_{CH2}, V_{CH3} are the segmental volumes of methine-, methylene-, and methyl groups and n_{CH}, n_{CH2}, n_{CH3} are the number of CH, CH_2, CH_3 groups per lipid molecule, respectively. A detailed description of the temperature dependence can be found in (5). Experimental and calculated molecular volumes were found to agree within experimental uncertainties (Table S2).

Estimation of temperature dependent compositional changes

Assuming that the inclination of tielines remains constant with temperature, tieline endpoints can be estimated using the lever rule. Specifically, we determined the distances between tieline midpoints and tieline endpoints $\overline{Ld}$, $\overline{Lo}$ using

$$c_{Ld}(T) = \frac{\overline{Lo}(T)}{\overline{tl}(T)} \tag{4}$$

$$c_{Lo}(T) = 1 - c_{Ld}(T) = \frac{\overline{Ld}(T)}{\overline{tl}(T)}, \tag{5}$$

where $\overline{tl}(T)$ is the overall tieline length. $\overline{tl}$ was estimated to change with temperature in proportion to the L_o fraction. Arbitrary functions were used to fit $c_{Lo}(T)$ data. Results are shown in Fig. S9. Estimates reproduce the asymmetric closing-in of the phase boundaries reported previously (6), which originate here from the measured increase of the L_d phase fraction.

Uppamoochikkal et al. (1) reported a small but significant increase of the tieline inclination angle with temperature. Therefore we checked, how this would influence our structural results. Note that this concerns only the area per lipid A, as results for all other parameter do not depend on knowing the specific lipid compositions of L_o/L_d domains. Hence, we tested several functions for the temperature behavior of the tielines, without finding any effects on the relative changes of A. Changes were observed for absolute values of A at the higher temperatures. However, they remained small, i.e. within ±3 %.

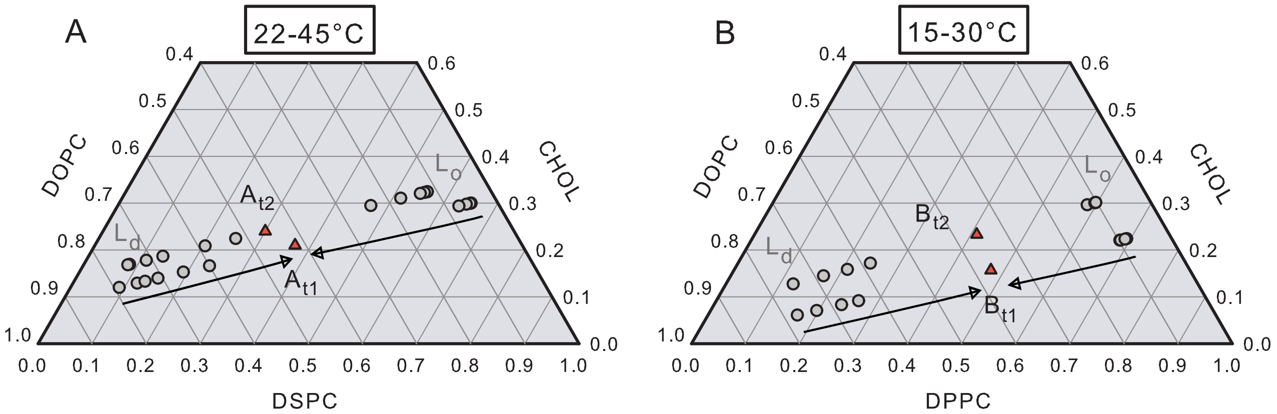

Figure S9: Estimated temperature dependence of tieline endpoints for DOPC/DSPC/CHOL (panel A) and DOPC/DPPC/CHOL (panel B). Triangles represent starting points at the tieline midpoints. Circles indicate the corresponding estimated tieline endpoints, which shift toward the midpoints as temperature is increased. The temperature increment was 5 ℃ only the first temperature step for DOPC/DSPC/CHOL was smaller (3 ℃). Note that L_d endpoints approach tieline midpoints more rapidly than L_o endpoints.

Supporting References

1. Uppamoochikkal, P., S. Tristram-Nagle, and J. F. Nagle, 2010. Orientation of tie-lines in the phase diagram of DOPC:DPPC:cholesterol model biomembranes. *Langmuir* 26:17363–17368.

2. Heberle, F. A., J. Wu, S. L. Goh, R. S. Petruzielo, and G. W. Feigenson, 2010. Comparison of three ternary lipid bilayer mixtures: FRET and ESR reveal nanodomains. *Biophys. J* . 99:3309–3318.

3. Hodzic, A., M. Rappolt, H. Amenitsch, P. Laggner, and G. Pabst, 2008. Differential modulation of membrane structure and fluctuations by plant sterols and cholesterol. *Biophys. J.* 94:3935–3944.

4. Greenwood, A. I., S. Tristram-Nagle, and J. F. Nagle, 2006. Partial molecular volumes of lipids and cholesterol. *Chem. Phys. Lipids* 143:1–10.

5. Koenig, B. W., and K. Gawrisch, 2005. Specific volumes of unsaturated phosphatidylcholines in the liquid crystalline lamellar phase. *Biochim. Biophys. Acta.* 1715:65–70.

6. Buboltz, J. T., C. Bwalya, K. Williams, and M. Schutzer, 2007. High resolution mapping of phase behavior in a ternary lipid mixture: Do lipid-raft phase boundaries depend on sample-prep procedure? *arXiv:0706.1374 [physics]* arXiv: 0706.1374.
