# Supplementary material for: In Situ Determination of Structure and Fluctuations of Coexisting Fluid Membrane Domains
Source: Biophys J. 2015 Feb 17;108(4):854–62. doi: 10.1016/j.bpj.2014.11.3488 (PMC4336378; doi:10.1016/j.bpj.2014.11.3488)
Supplement: Document S2. Article plus Supporting Material [file mmc2.pdf]

Article

In Situ Determination of Structure and Fluctuations of Coexisting Fluid Membrane Domains

Peter Heftberger,[1,2] Benjamin Kollmitzer,[1,2] Alexander A. Rieder,[1,2] Heinz Amenitsch,[3] and Georg Pabst[1,2,*]
[1]Biophysics Division, Institute of Molecular Biosciences, University of Graz, NAWI Graz, Graz, Austria; [2]BioTechMed-Graz, Graz, Austria; and [3]Institute of Inorganic Chemistry, Graz University of Technology, Graz, Austria

ABSTRACT Biophysical understanding of membrane domains requires accurate knowledge of their structural details and elasticity. We report on a global small angle x-ray scattering data analysis technique for coexisting liquid-ordered (L_o) and liquid-disordered (L_d) domains in fully hydrated multilamellar vesicles. This enabled their detailed analysis for differences in membrane thickness, area per lipid, hydrocarbon chain length, and bending fluctuation as demonstrated for two ternary mixtures (DOPC/DSPC/CHOL and DOPC/DPPC/CHOL) at different cholesterol concentrations. L_o domains were found to be ~10 Å thicker, and laterally up to 20 Å^2/lipid more condensed than L_d domains. Their bending fluctuations were also reduced by ~65%. Increase of cholesterol concentration caused significant changes in structural properties of L_d, while its influence on L_o properties was marginal. We further observed that temperature-induced melting of L_o domains is associated with a diffusion of cholesterol to L_d domains and controlled by L_o/L_d thickness differences.

INTRODUCTION

Since the formulation of the raft model in 1997 by Simons and Ikonen (1) significant scientific efforts have been devoted to the characterization of physical properties of liquid-disordered L_d and liquid-ordered L_o domains (2–11). Membrane rafts are thought to be small (nanoscopic) and highly dynamic platforms enriched in sphingolipids and cholesterol, enabling diverse cellular functions, but have so far escaped any direct visualization in live cells (5,12). Hence, the existence of rafts remains a highly controversial issue. For example Frisz et al. (13,14), using secondary ion mass spectrometry on fibroblasts, observed sphingolipid domains, in which cholesterol was evenly distributed throughout the membrane, thus challenging the standard raft hypotheses.

In contrast to natural membranes, domains in lipid-only systems can grow up to several micrometers in size, enabling their detection (e.g., by optical microscopy (15)) and study with respect to the physics pertaining to their stability, size, or effect on protein sorting, to name but a few examples (8). One of the parameters involved in, e.g., protein sorting, is the difference in thickness between the L_o and L_d domains and the corresponding match to the protein's transmembrane region (see, e.g., Killian (16) and Pabst (17)).

To address these issues, diverse experimental and theoretical techniques have been employed to explore structural and elastic properties of L_o/L_d phases (see, e.g., the literature (18–32)). Scattering experiments are of particular interest in this respect, because they allow for a label-free determination of membrane structure and dynamics (33). However, contrast between L_o and L_d domains is low. This can be addressed, for example, by contrast variation, using neutron scattering (34). In recent years, this technique has been used largely by Katsaras and coworkers, showing, e.g., the coupling of domain size and membrane thickness mismatch between L_o and L_d (35).

Alternatively, early x-ray experiments used Triton X-100 (Dow Chemical, Midland, MI) to separate detergent-resistant from detergent-soluble membranes, respectively (22). However, the application of detergents on membranes may adversely influence the mixing behavior of membrane lipids (36), limiting the applicability of this approach.

Another possibility, which is being explored in this work, makes use of the experimental finding that macroscopic domains are typically in registry in multilamellar systems (see, e.g., Chen et al. (28), Tayebi et al. (37), and Karmakar et al. (38)), meaning: L_o and L_d domains form lamellar lattices with distinct Bragg peaks. The challenges to be met here are 1) overlapping L_o/L_d Bragg reflections, in particular at low scattering angles; and 2) the small number of solid orders (only 2–3) displayed by L_d phases in fully hydrated multilamellar vesicles (MLVs), limiting the structural information content when only Bragg peak intensities are analyzed (39).

The latter issue is particularly well known for single-phase fluid bilayers, and has led to the development of a global SAXS data analysis technique that takes into account both Bragg peaks and diffuse scattering (39). Most recently,

Submitted October 16, 2014, and accepted for publication November 24, 2014.

*Correspondence: georg.pabst@uni-graz.at

This is an open access article under the CC BY license (http://creativecommons.org/licenses/by/4.0/).

Editor: Anne Kenworthy.

© 2015 The Authors
0006-3495/15/02/0854/9 $2.00

http://dx.doi.org/10.1016/j.bpj.2014.11.3488

we have advanced the technique by incorporating the scattering density profile (SDP) model (40), enabling us to determine membrane structure and bending fluctuations from homogeneous MLVs at high resolution (41).

To access coexisting fluid domains in MLVs, the global SAXS data analysis needs to be further extended. This was achieved in this work by assuming a linear combination of scattering intensities originating from L_o and L_d phases. The method was applied to two ternary mixtures, with the high-melting lipids DPPC (dipalmitoylphosphatidylcholine) or DSPC (distearoylphosphatidylcholine), the low-melting lipid DOPC (dioleoylphosphatidylcholine), and CHOL (cholesterol). Summaries of the studied samples and applied nomenclature are given in Fig. 1 and Table S1 in the Supporting Material.

We observed distinct structural and elastic properties of L_o and L_d domains as a function of temperature and composition (lipid chain length and cholesterol concentration).

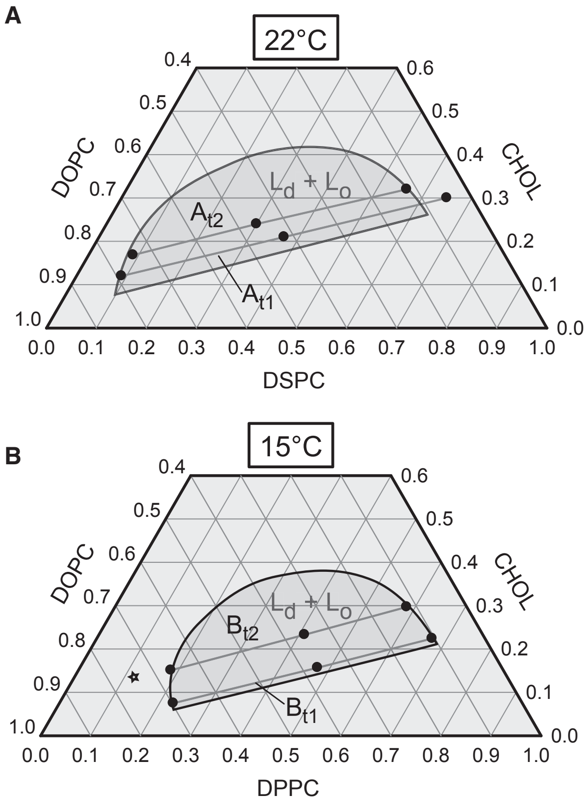

FIGURE 1 Overview of samples studied in this work. (*Solid circles*) Their location is shown in previously reported compositional phase diagrams of (*A*) DOPC/DSPC/CHOL (50) and (*B*) DOPC/DPPC/CHOL (49). In fluid-fluid phase coexistence regions (*dark-shaded areas*), demixing into L_o and L_d domains occurs along tielines. Two tielines for each system (A_{t1}, A_{t2} and B_{t1}, B_{t2}) with three different compositions at the L_d, L_o endpoints and the tieline center were studied (see Table S1 for detailed lipid composition). Note that tieline endpoints for L_o on A_{t1} and L_d on B_{t2} (*star*) are outside previously reported phase boundaries. This is either due to updates in phase boundaries for isolated tielines (A_{t1} (35)) or experiments performed in this study (B_{t2}, see Results).

Most interestingly, we found that increased cholesterol concentrations reduce the thickness difference between L_d and L_o domains, which leads to a decrease of line tension and in turn promotes the temperature induced melting of L_o domains.

MATERIALS AND METHODS

Sample preparation

DPPC, DSPC, and DOPC were purchased from Avanti Polar Lipids (Alabaster, AL), and cholesterol was obtained from Sigma-Aldrich (Vienna, Austria). All lipids were used without further purification, with all chemicals being of professional analysis quality. Lipid stock solutions were prepared by dissolving weighted amounts of dry lipid in chloroform/methanol (2:1, v/v) and then mixed at appropriate ratios (see Table S1 for all samples and their corresponding compositions). Subsequently, lipid solutions were dried under a stream of nitrogen and then placed under vacuum for ~12 h, forming a thin lipid film on the bottom of glass vials. Dry films were hydrated using 18 MΩ/cm water and incubated for 6 h above the main transition temperature of the high-melting lipid (DPPC or DSPC), repetitively cycling vortex-mixing and freeze-thaw procedures using liquid N_2. The final lipid concentration for each sample was 50 mg/mL. All samples were prepared at least twice (with a time delay of several weeks) starting from pure lipid powders to check for reproducibility. Lattice constants (d-values) varied by $< \pm 0.5\%$. Furthermore, thin layer chromatography on randomly selected samples revealed no decomposition of the samples into lyso lipids or free fatty acids.

Small angle x-ray scattering

X-ray scattering data were acquired at the Austrian SAXS beamline Elettra Trieste, Trieste, Italy, using 8 keV photons. Diffraction profiles were detected utilizing a Mar300-image-plate detector (MarResearch, Norderstedt, Germany) and calibrated using silver behenate. Lipid dispersions were taken up in 1-mm-thick quartz capillaries and inserted into a multiposition sample holder. All samples were equilibrated for a minimum of 10 min before measurement using a circulating water bath. The exposure time was set to 30 s. Scattering patterns were integrated using the program FIT2D (42). Background scattering originating from capillaries, water, and air was subtracted (43), and data sets were normalized using the transmitted intensity, which was measured by a photodiode placed in the beam stop.

Analysis of coexisting domains

To analyze the scattering profile of MLVs exhibiting L_o/L_d phase coexistence, we adopted the full-q-range model by Heftberger et al. (41) for homogeneous bilayers. For the latter systems, the scattered intensity is given by

$$I(q) = \frac{1}{q^2}\left[|F(q)|^2 S(q)\left(1 - N_{\mathrm{diff}}\right) + \left|F(q)^2\right| N_{\mathrm{diff}}\right], \quad (1)$$

where $q = 4\pi \sin\theta/\lambda$ is the scattering vector, λ is the wavelength, 2θ is the scattering angle relative to the incident beam, and N_{diff} is the diffuse scattering originating from positionally uncorrelated bilayers. The structure factor $S(q)$ is given by the Caillé theory (39,44–46), yielding access to bending fluctuations via the Caillé parameter

$$\eta \propto \frac{T}{\sqrt{K_C B}},$$

with K_C as the bilayer bending rigidity and B as the bulk modulus of interactions (39). The form factor $F(q)$ is the Fourier transform of the electron density profile of a bilayer, described in terms of the SDP model (40).

Neglecting putative cross-correlations between L_o and L_d domains, the scattered intensity of coexisting fluid domains can be modeled by a linear combination of the individual L_o/L_d intensities,

$$I(q) = \frac{1}{q^2}\left[\left(1 - N_{\text{diff}}\right)I_{\text{corr}}(q) + N_{\text{diff}}I_{\text{uncorr}}(q)\right], \quad (2)$$

where

$$I_{\text{corr}}(q) = |F_{Ld}(q)|^2 S_{Ld}(q)c_{Ld} + |F_{Lo}(q)|^2 S_{Lo}(q)(1 - c_{Ld})$$

and

$$I_{\text{uncorr}}(q) = |F_{Ld}(q)|^2 c_{Ld} + |F_{Lo}(q)|^2 (1 - c_{Ld})$$

are the scattering intensities originating from positionally correlated and uncorrelated bilayers, respectively. The subscripts Lo and Ld denote the individual contributions of the domains to $S(q)$ and $F(q)$, and c_{Ld} refers to the L_d phase fraction in the sample. Analysis of the scattered intensity of coexisting phases in terms of this model yields bilayer structural parameters and bending fluctuations simultaneously for L_o and L_d. A strict requirement for its application is that domains are in registry in the direction normal to the bilayer plane, meaning: two distinct lamellar lattices need to be observed. This is typically the case for macroscopic domains, observed for example when diunsaturated lipids such as DOPC are used as low-melting membrane component in ternary raftlike mixtures (28,37,38).

The SDP model, used to describe the form factors, parses the bilayer lipids into quasi-molecular fragments and calculates their volume probability distributions. The model was originally designed for determining single lipid component bilayers (40,47). Pan et al. (48) extended the SDP analysis to binary lipid mixtures containing cholesterol. To this end the contribution of cholesterol was merged with that of methylene (CH_2) groups, which was recently also applied successfully to homogenous MLVs (41). Because our studied L_o and L_d domains contain different amounts of three lipids, effective lipid molecules were constructed for the SDP description, by first merging the contributions from the unsaturated and saturated lipids and then adding cholesterol to the CH_2 regime as described above. Saturated and unsaturated lipids differ with respect to the number of CH_2 and methine (CH) groups. Due to the absence of scattering contrast between CH and CH_2 for x-rays, these groups can be merged (40,41). Our final parsing approach consisted of five groups for each phase, composed of the following: 1) $CholCH_3$ (Choline methyl), 2) PCN (Phosphate + CH_2CH_2N), 3) CG (Carbonyl + glycerol) groups, 4) CH_2, and 5) CH_3 methyl groups at the bilayer center.

This approach is further justified by its compatibility to previously reported molecular dynamics (MD) data (31), as demonstrated in Fig. 2. For details of the functional forms used to describe the individual groups, we refer to the literature (40,41,48). As detailed previously (41), a genetic algorithm was applied for fitting the global model to experimental data.

Membrane structural parameters such as hydrocarbon chain length d_C, Luzzati thickness d_B, water layer thickness d_W, and the area per lipid $A = 2V_L/d_B$ were defined and calculated from the SDP profiles as described in Heftberger et al. (41). V_L is the total lipid volume, which is assumed to be given by the molecular-weighted average

$$V_L(T) = \sum_i x_i(T)V_i(T), \quad (3)$$

where x_i values are the lipid molar ratios in L_d and L_o taken from Uppamoochikkal et al. (49) and Heberle et al. (50), V_i values are the corresponding molecular lipid volumes, and T is the temperature. Temperature-dependent V_i values were calculated according to the method of Koenig and Gawrisch (51), and the volume of cholesterol within lipid bilayers was taken to be

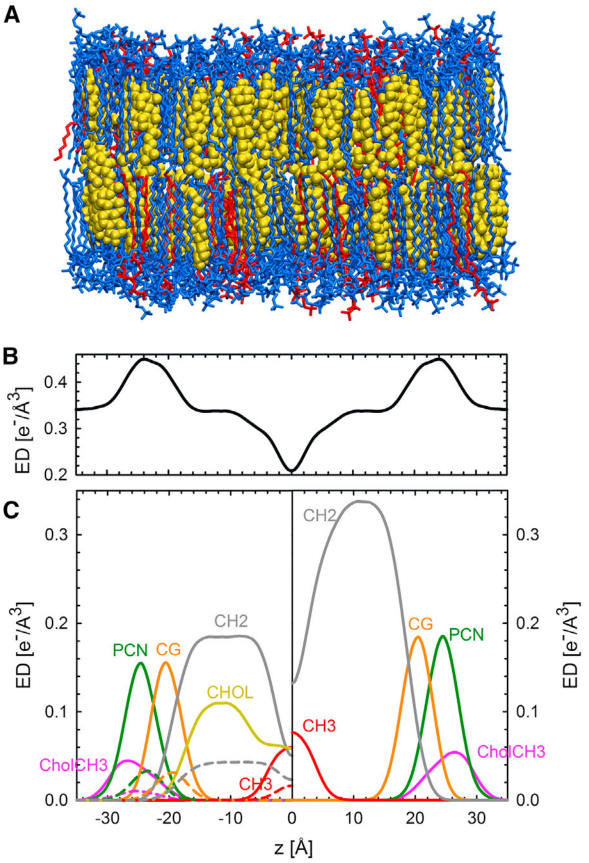

FIGURE 2 Parsing scheme of ternary lipid mixtures based on MD simulations of an L_o phase of DOPC/DPPC/CHOL (31). (*A*) Snapshot of the equilibrated system. DPPC lipids, *blue*; DOPC, *red*; cholesterol, *yellow*. (*B*) Calculated electron density profile. (*C*) Electron densities of molecular groups, calculated using SIMTOEXP (70). (*Left*) Individual contributions of DPPC (*solid lines*) and DOPC (*dashed lines*) for the $CholCH_3$, PCN, CG, CH_2, and CH_3 groups. (*Yellow line*) Contribution of cholesterol. (*Right*) Condensed parsing scheme after merging individual contributions as detailed in the main text. To see this figure in color, go online.

630 Å^3 (52). Calculated tieline endpoint V_L were in good agreement (<2%) with experimental values determined by dilatometry (Supporting Material). Note that dilatometry yields a globally averaged value for the lipid volume and is thus not able to discern between L_o and L_d in the phase coexistence regime. Thus experimental V_L can be obtained for tieline endpoints, only. The temperature dependence of x_i was estimated by the lever rule using the experimentally determined L_d and L_o fractions (c_{Ld} and $1 - c_{Ld}$), assuming 1) that the inclination of the tieline remains constant, and 2) that the tieline length changes according to the L_o fraction with temperature (Supporting Material).

RESULTS AND DISCUSSION

Establishing the global analysis for two phases

Our strategy to validate this analysis was as follows. 1) We evaluated tieline endpoint SAXS data. At endpoints, either L_o or L_d should exist as a single phase, thus allowing application of our previous analysis for homogeneous MLVs (41). 2) The phase coexistence model was applied to a

composition close to the tieline midpoint and the achieved results were compared to endpoint data.

Phase boundaries, in particular, are subject to considerable uncertainties (8,49,53–55), whereas recent tieline orientation data are considered to be more reliable. In this study, we applied previously published compositional phase diagrams from Heberle et al. (50) and Uppamoochikkal et al. (49). Note that Uppamoochikkal et al. (49) used the phase diagram reported by Veatch et al. (54), and constructed tielines using x-ray scattering on oriented lipid films.

Our measurement strategy allowed for an independent check of these data, as follows: 1) Bragg peaks of all samples measured per tieline are required to overlap, if tieline orientation is correct (49); and 2) only a single lamellar lattice should be observed at the phase boundaries.

For all samples, including replicas, L_o/L_d peak positions for tieline midpoints and endpoints matched, reassuring not only tieline orientation data, but also our sample preparation. The L_d endpoints of DOPC/DPPC/CHOL contained significant residual scattering from an L_o phase, revealing errors in the reported phase boundary. However, because L_o peaks overlapped with those of the L_o endpoint, we were able to subtract the L_o contribution (Fig. S1 in the Supporting Material). In independent experiments, using a laboratory x-ray camera, we determined for B_{t2} a new L_d endpoint by measuring several samples along the tieline until the L_o contributions vanished. The new endpoint composition is 0.748/0.124/0.128 (Fig. 1); corresponding SAXS data are shown in Fig. S2.

Fig. 3 details the results and analysis of the A_{t1} tieline. The L_d endpoint in this study showed some residual L_o contribution. However, it was small enough to be neglected. Global fits to tieline endpoints and midpoints show that our model is able to capture both the single-phase and two-phase coexistence, respectively. Insets to Fig. 3 show the volume probability distributions of individual quasi-molecular groups (see previous section) for L_o, L_d endpoints and the resulting electron density (ED) profiles for the coexisting case. The absolute ED in the hydrocarbon chain region of the L_o phase is significantly higher than in L_d. This can be explained by the higher amount of cholesterol in the L_o phase, with substantial ED contributions from the sterol ring.

All structural parameters for coexisting domains agreed remarkably to tieline endpoints (Tables 1 and S2), thereby validating our analysis. For example, the membrane thickness d_B for L_d and L_o endpoints is 37.5 and 49.7 Å, respectively. This compares well to $d_B = 38.1$ and 48.6 Å for L_d and L_o in the coexistence regime, meaning: differences are within <1 Å. Note that height differences Δd_B between L_d and L_o domains are in the same range as those reported between detergent-resistant and detergent-soluble membranes (22).

The two-phase analysis was further tested by checking whether a decrease of the hydrocarbon chain length of the

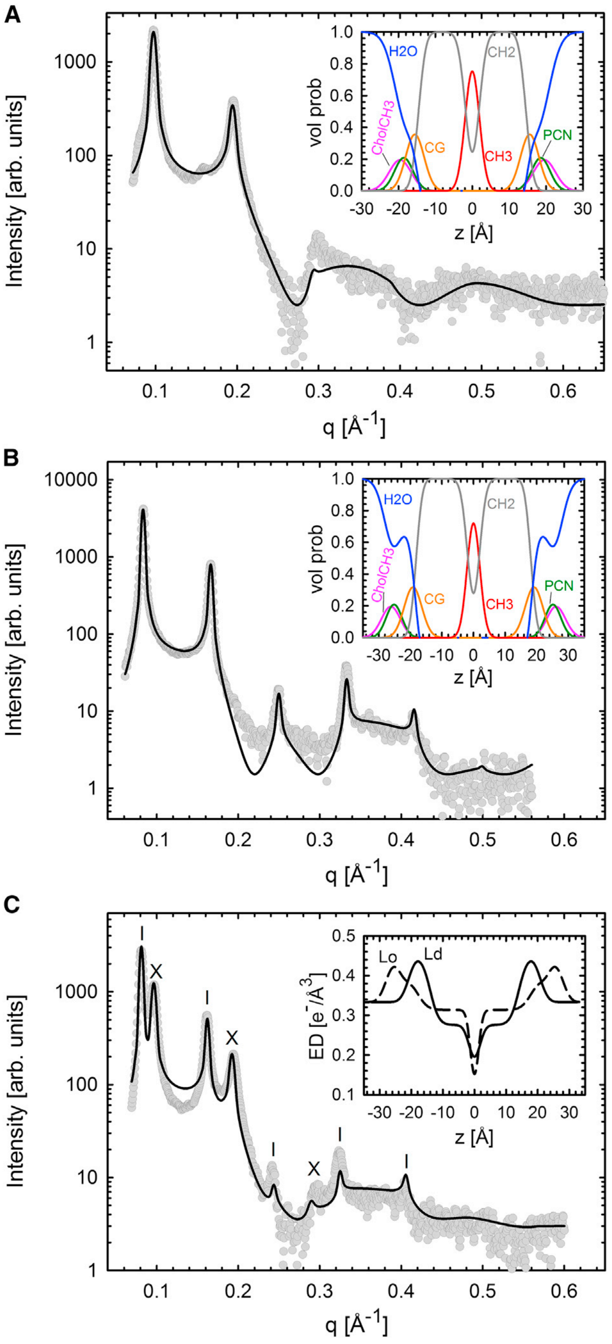

FIGURE 3 Validation of the global analysis for two coexisting phases for the A_{t1} tieline ($T = 22°$C). (*A* and *B*) Fits to L_d and L_o endpoint data, respectively. (*Insets*, both panels) Derived volume probability distributions. (*C*) Best fit to SAXS data at the A_{t1} tieline midpoint. Bragg reflections of L_o (*dashes*) and L_d (*crosses*) domains. (*Inset*) ED profiles for L_o and L_d phases. To see this figure in color, go online.

mixture's high-melting lipid leads to reasonable changes in domain structure. Exchanging DSPC with DPPC affected mainly the structure of the L_o phase. Picking for example the $t1$ tielines, d_B decreased by 2.6 Å, whereas only a minor decrease of 0.6 Å was found for L_d. Similar changes were found for other tielines, including d_C-values (Table 1).

TABLE 1 Structural results and bending fluctuations for coexisting L_d/L_o domains

Term	d_B	A	d_W	d_C	η
$A_{t1}(L_d)$	38.5	63.1	26.6	14.5	0.091
$A_{t1}(L_o)$	49.8	43.2	27.6	18.8	0.030
$A_{t2}(L_d)$	39.2	60.3	26.5	14.9	0.092
$A_{t2}(L_o)$	49.2	43.1	26.8	18.5	0.029
$B_{t1}(L_d)$	37.9	64.9	26.0	14.2	0.074
$B_{t1}(L_o)$	47.2	44.4	25.1	17.7	0.021
$B_{t2}(L_d)$	38.9	61.2	25.8	14.7	0.068
$B_{t2}(L_o)$	46.4	43.5	23.3	17.3	0.024

Parameter uncertainties are <2%.

Our findings are in excellent agreement with tieline orientation (Fig. 1); because the high-melting lipid is located in L_o domains, we observe a thinning by exchanging DSPC to DPPC, which contains two CH_2 groups less per acyl chain. Kučerka et al. (47) reported a similar thickness difference for pure DSPC and DPPC bilayers. L_d phases contain mainly DOPC and are consequently barely effected by the lipid exchange. Further structural parameters for L_d (Table 1) are close to that of pure DOPC (40).

Finally, we compare areas per lipid, A, which differ significantly between L_d and L_o phases (Table 1). The L_d domains exhibited A values between 60 and 65 Å^2, which is in the range of values reported for fluid single lipid bilayers (40,47). Areas are ~20 Å^2 smaller for L_o domains. The main reason for this difference is the condensing effect of cholesterol, which was previously reported for several binary phosphatidylcholine/cholesterol mixtures (48,56–58) and is now also observed for coexisting L_o/L_d domains. Another manifestation of this ordering effect is the decrease of the bending fluctuation parameter from η ~ 0.08 (L_d) to ~0.03 (L_o).

Thus, concluding this section: our global SAXS data analysis yields, within typical uncertainties of the SDP model, robust high-resolution results for structure and fluctuations of coexisting L_o/L_d domains. This allows us to obtain reliable insights on changes of these parameters, e.g., as a function of composition or temperature. Results of such experiments are presented in the following sections.

Effect of cholesterol on domains

To study the influence of raising cholesterol concentration, we compare the $t1$ and $t2$ tieline midpoints for both ternary mixtures. Scattering profiles and fits are plotted in Fig. 3 (Figs. S3, S4, and S5), while results for structural and elastic parameters are presented in Table 1. For both systems studied, d increased by ~0.75 Å for L_d domains, but decreased by ~2 Å for L_o upon increasing cholesterol content. Our analysis revealed that the increase of d for L_d is mainly due to a thickening of its bilayer, whereas only approximately one-third of the decrease of d for L_o can be attributed to d_B. A decrease of d_B for L_o upon increasing cholesterol concentration may seem counterintuitive, but the marginal additional ordering effect due to more cholesterol is overcompensated by a reduction of the high-melting lipid concentration. Most of the change in d for L_o is due to a decrease of the interstitial water layer (1–2 Å), which may originate either from an increase in net attractive forces, or a decrease in net repulsive forces between L_o domains. This effect cannot be attributed to an increased bending rigidity due to the higher cholesterol content (59), because the fluctuations did not decrease (Table 1). Instead, a decrease of hydration or an increase of van der Waals forces might be the reason.

The area per lipid was found to be smaller for L_d domains of the $t2$ tielines (Table 1), which can be attributed to the well-known condensing effect of cholesterol (58). For L_o domains, changes for A were found to be insignificant (within experimental uncertainty). However, it is interesting to note that the variation of A for L_o even across DOPC/DPPC/CHOL and DOPC/DSPC/CHOL is within $< \pm 2\%$. This indicates that the average value A ~ 43.6 Å could be the tightest possible packing of lipids in the L_o phase. More structural data on L_o would certainly be needed to validate this notion.

Temperature dependence of L_o/L_d domains

Starting from the reported compositional phase diagrams (Fig. 1), we increased temperature in steps of 5°C until we reached a homogeneous phase. The transition is observed as a merging of the L_o and L_d lattices into a single L_d phase lattice (Fig. 4). For DOPC/DSPC/CHOL, the transition at T_C occurred between 45 and 50°C, and for DOPC/DPPC/CHOL, between 30 and 35°C. Note that our temperature resolution does not allow us to determine T_C with high accuracy. Fig. 5 A compares the results for d_B of A_{t1} and B_{t1}. The L_o phase of B_{t1} was found to be 3 Å thinner than that of A_{t1}. Because changes with temperature are similar for both $t1$ and $t2$ tielines (Figs. 5 and S6), we can therefore limit the discussion to the $t1$ tielines. In the temperature range of 22–45°C d_B of L_d domains increased monotonously by ~1–1.5 Å, whereas d_B for L_o decreased at the same time by 2 Å. Close to T_C, these changes are significantly accelerated. Above the transition temperature, d_B is similar to that of the L_d phase just below T_C. Interestingly, d_B is approximately equal for DSPC- and DPPC-containing samples above T_C, including the $t2$ tielines (Fig. S6) despite the difference in hydrocarbon chain length.

The thickness of single-phase fluid lipid bilayers typically decreases with increasing temperature (47,60,61). Thus, the thickening of the L_d domains upon approaching T_C from below is surprising. Davis and Schmidt (23) recently suggested, based on NMR data, that the cholesterol fraction in L_o decreases with temperature. Consequently, L_d would get enriched in cholesterol. Because of the associated condensation effect of cholesterol, one would then expect

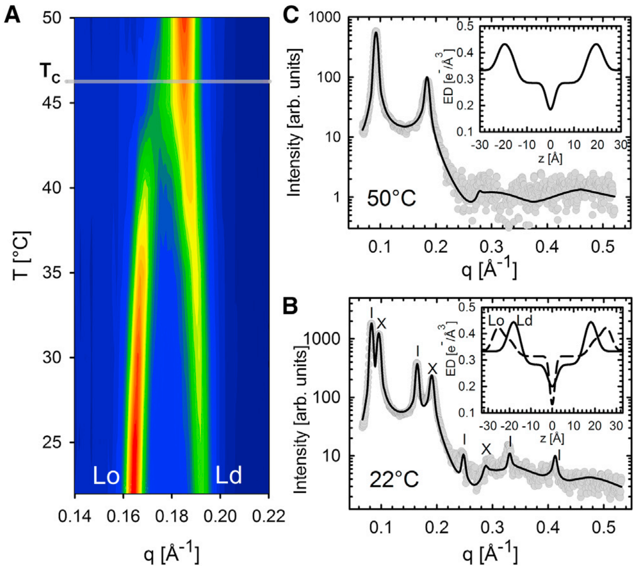

FIGURE 4 Temperature behavior of DOPC/DSPC/CHOL (tieline $t2$) as revealed by SAXS. (*A*) Contour plot of the second-order Bragg reflections indicated as L_o and L_d. Note that the smooth appearance of data is due to an interpolation procedure between the individual frames. The critical temperature T_C is between 45 and 50°C. At $>T_C$, only a single lamellar lattice is observed. (*B*) Measured scattering at 22°C with the indicated Bragg reflections for L_o (*dashes*) and L_d (*crosses*) domains. (*C*) The same, for 50°C. (*Solid lines*) Best fits. (*Insets*, both panels) Resulting ED profiles for L_o and L_d phases. To see this figure in color, go online.

an increase of the L_d domain thickness. Our results consequently corroborate this scenario.

The area per lipid is inversely proportional to d_B. Hence the temperature changes of A are similar to d_B, but just with inverted trends, i.e., A decreases for L_d and increases for L_o, as observed in Fig. 5 *B*. We further note that areas above T_C are alike for all systems and tielines studied, which appears reasonable in view of the similar A-values reported for single-component DPPC and DSPC membranes (47). Changes of the water layers in turn appear to be decoupled from the trends of d_B and A. We found a general increase of d_W for L_d domains and a decrease for L_o domains (Fig. 5 *C*) below T_C, with changes close to T_C being more pronounced for the L_o phase. These findings are not straightforward to explain, in particular because the Caillé parameter did not show a strong increase of bending fluctuations for L_d, or decrease for L_o, respectively. Instead, an overall decrease in η was found for L_d (Fig. 6) and an increase in the vicinity of T_C for L_o. These two trends can be explained by the temperature-driven diffusion of cholesterol to L_d, as discussed above. Specific changes in d_W in turn appear to be caused by other influences on intermembrane interactions. Additional experiments, such as a combination of SAXS with osmotic stress (62), are needed to address this issue properly.

Melting of L_o domains can be further assessed by c_{Ld}, corresponding to the L_d phase fraction (Eq. 2). All studied systems show a steady increase of c_{Ld} as T_C is approached from

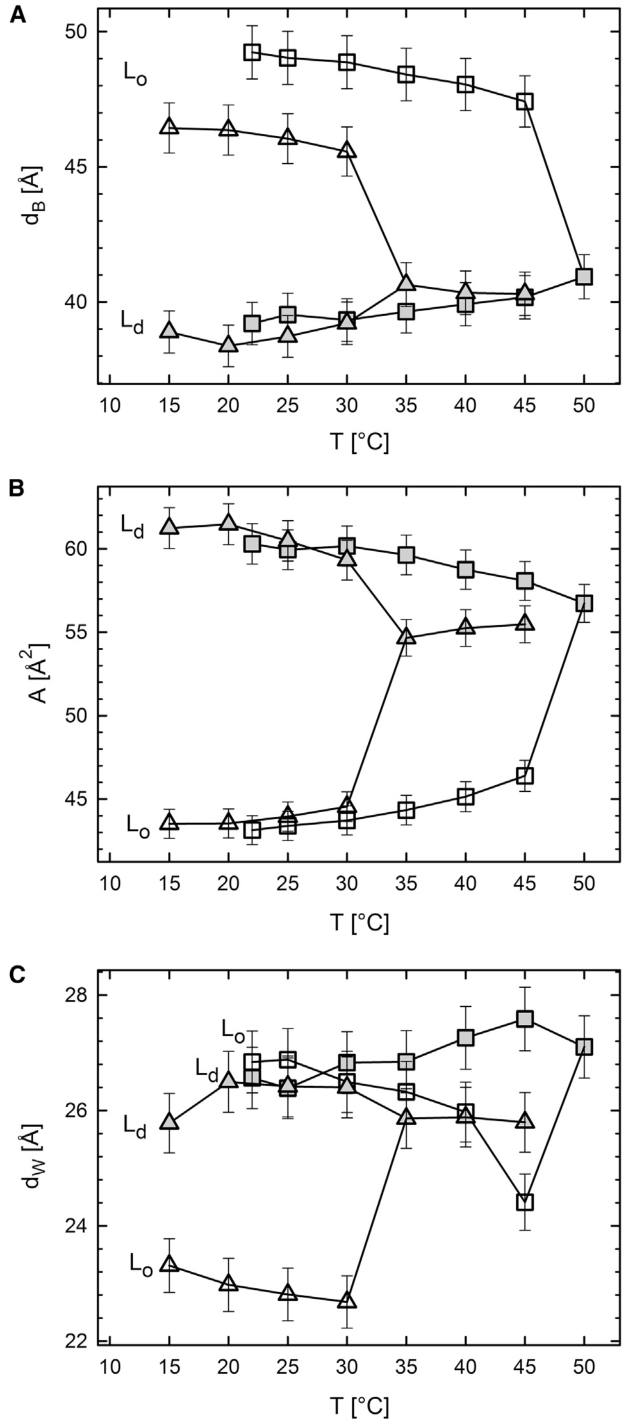

FIGURE 5 Temperature dependence of structural parameters of DOPC/DSPC/CHOL (*squares*) and DOPC/DPPC/CHOL (*triangles*), starting from the $t1$ tieline midpoints (Fig. 1). (*A*) Bilayer thickness, (*B*) area per lipid, and (*C*) water layer thickness, for L_d (*solid symbols*) and L_o (*open symbols*) domains as a function of temperature.

below, while the $t2$ composition of DOPC/DSPC/CHOL exhibited the largest overall L_d fraction (Fig. 7). The increase of c_{Ld} also signifies that the L_d tieline endpoints approach the chosen midpoints more rapidly than the L_o endpoints.

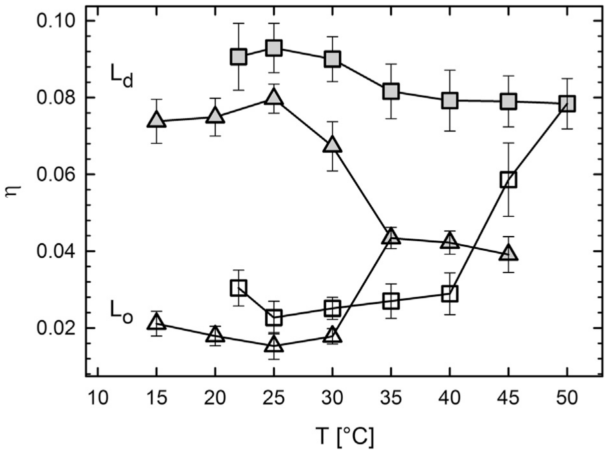

FIGURE 6 Temperature dependence of bending fluctuations of coexisting DOPC/DSPC/CHOL (*squares*) and DOPC/DPPC/CHOL (*triangles*) domains, for $t1$ tieline compositions (Fig. 1 and Table S1). (*Shaded symbols*) Results for L_d domain; (*open symbols*) results for L_o domain.

Hence, the L_o/L_d coexistence regime reduces asymmetrically with temperature, i.e., closes-in faster on the L_d boundary than on the L_o boundary, in agreement with Buboltz et al. (63) (and see the Supporting Material).

Finally, it is interesting to compare the relative increase of the L_d fraction in the studied temperature range. For DOPC/DSPC/CHOL, c_{Ld} increased with temperature by 16% for the $t1$ and 25% for the $t2$ tieline, respectively. Differences are smaller for DOPC/DPPC/CHOL, with $\Delta c_{Ld} = 9\%$ for $t1$ and 16% for $t2$, but here changes are more pronounced at higher cholesterol content. Hence, increasing cholesterol concentration appears to promote melting of L_o. This can be understood by reviewing the height differences between L_d and L_o domains (Table 1). For DSPC-containing mixtures, $\Delta d_B = 11.3$ Å for the $t1$ tieline and $\Delta d_B = 10$ Å or $t2$, whereas $\Delta d_B = 9.7$ Å and $\Delta d_B = 7.5$ Å for the $t1$ and $t2$ tielines in DOPC/DPPC/CHOL. Thus, Δd_B decreases with cholesterol concentration for both systems. The height differences are

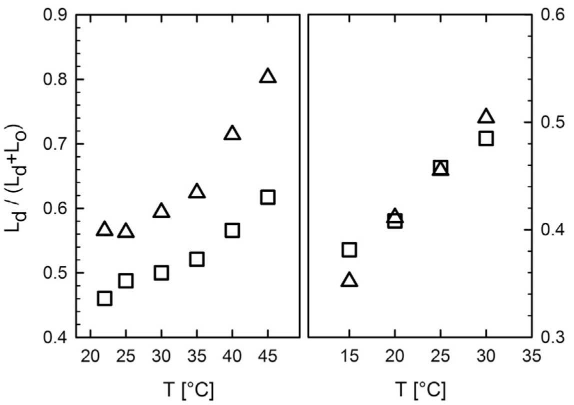

FIGURE 7 Variation of L_d phase fraction with temperature. (*Left*) Results for DOPC/DSPC/CHOL. (*Right*) Results for DOPC/DPPC/CHOL. (*Squares*) Results for $t1$ tielines; (*triangles*) results for $t2$ tielines.

related to the line tension γ between L_d and L_o domains. In particular, Akimov et al. (30) showed that $\gamma \propto \Delta d_B^2$. Consequently, the cholesterol-induced decrease of Δd_B leads to a lowering of γ, facilitating the melting of L_o domains.

CONCLUSIONS

We introduced a global SAXS data analysis technique, which yields structural and elastic properties of coexisting L_o/L_d domains in multilamellar vesicles. The model captures 1) high structural resolution by incorporating the SDP model (40) and 2) bending fluctuations through a Bragg peak line-shape analysis in terms of the Caillé theory structure factor (39). The method has been verified on DOPC/DSPC/CHOL and DOPC/DPPC/CHOL mixtures by comparing tieline endpoint with midpoint data of corresponding phase coexistence samples, and by essaying whether it captures the effects of chain length increase for L_o domains, such as thickness increase or a decrease of lipid area.

We further characterized two tielines for each ternary mixture to study effects of increased cholesterol concentration. Interestingly, additional cholesterol affected mostly structural properties of the L_d phase (increase of d_B and d_C, decrease of A, and decrease of η), whereas L_o appeared to be already saturated. A further effect of higher cholesterol concentration was a decrease of the thickness difference between L_o and L_d domains, leading to a lowering of line tension and consequently to a destabilization of L_o domains that is somehow analogous to the well-known order/disorder effect of cholesterol in binary lipid mixtures (64). The temperature behavior revealed structural and elastic changes during melting of L_o domains, which suggest that cholesterol diffuses into L_d domains even below T_C.

Because of its ability to analyze phase coexistence data without using labels, our technique should be able contribute to resolving several open questions in the field. One of the many controversial issues of raftlike lipid mixtures, for example, is their critical behavior across the transition into a homogeneous phase. According to theory, any defined order parameter should vary $\propto (T_C - T)^{\beta}$, where the critical exponent β is either 0.125 or 0.325, depending on whether the system follows the two- or three-dimensional Ising model, respectively (23,65–68). Fluorescence microscopy experiments on compositional fluctuations in the vicinity of T_C revealed a two-dimensional Ising model-like behavior (65–67). In contrast, atomic force measurements on the height-difference of L_o/L_d (68) and first momenta of NMR spectra (23) reported critical exponents favoring the three-dimensional Ising model. We analyzed the height difference between L_o and L_d phases across T_C to determine a critical exponent (Fig. S8). Our results favor the two-dimensional Ising model, but the apparently coarse temperature steps preclude us from any firm statement.

Future studies will be designed to exactly address this issue. Another interesting application for our technique will be to predict protein activity and partitioning in domains (17), which can be achieved by adding information on spontaneous curvatures (69) and bending elasticities (31,33) of L_o and L_d domains. This work is underway in our laboratory.

SUPPORTING MATERIAL

Supporting Materials and Methods, nine figures, and two tables are available at http://www.biophysj.org/biophysj/supplemental/S0006-3495(14)04817-6.

ACKNOWLEDGMENTS

We are grateful to George Khelashvili and Milka Doktorova for sharing MD simulation data on L_o phases and for providing us with the MD simulation snapshot. We further thank Frederick A. Heberle, Jianjun Pan, and John Katsaras for valuable discussions.

This work was supported by the Austrian Science Fund FWF, Project No. P24459-B20 (to G.P.).

REFERENCES

1. Simons, K., and E. Ikonen. 1997. Functional rafts in cell membranes. *Nature.* 387:569–572.
2. Simons, K., and D. Toomre. 2000. Lipid rafts and signal transduction. *Nat. Rev. Mol. Cell Biol.* 1:31–39.
3. Edidin, M. 2003. The state of lipid rafts: from model membranes to cells. *Annu. Rev. Biophys. Biomol. Struct.* 32:257–283.
4. McMullen, T. P. W., R. N. A. H. Lewis, and R. N. McElhaney. 2004. Cholesterol-phospholipid interactions, the liquid-ordered phase, and lipid rafts in model and biological membranes. *Curr. Opin. Colloid Interface Sci.* 8:459–468.
5. London, E. 2005. How principles of domain formation in model membranes may explain ambiguities concerning lipid raft formation in cells. *Biochim. Biophys. Acta.* 1746:203–220.
6. Brown, D. A., and E. London. 2000. Structure and function of sphingolipid- and cholesterol-rich membrane rafts. *J. Biol. Chem.* 275:17221–17224.
7. Brown, D. A., and E. London. 1998. Structure and origin of ordered lipid domains in biological membranes. *J. Membr. Biol.* 164:103–114.
8. Goñi, F. M., A. Alonso, ..., J. L. Thewalt. 2008. Phase diagrams of lipid mixtures relevant to the study of membrane rafts. *Biochim. Biophys. Acta.* 1781:665–684.
9. Marsh, D. 2010. Liquid-ordered phases induced by cholesterol: a compendium of binary phase diagrams. *Biochim. Biophys. Acta.* 1798:688–699.
10. Marsh, D. 2009. Cholesterol-induced fluid membrane domains: a compendium of lipid-raft ternary phase diagrams. *Biochim. Biophys. Acta.* 1788:2114–2123.
11. Heberle, F. A. 2014. Liposome-based models for membrane rafts: methodology and applications. *In* Liposomes, Lipid Bilayers and Model Membranes: From Basic Research to Application. CRC Press, Boca Raton, FL.
12. van Meer, G., D. R. Voelker, and G. W. Feigenson. 2008. Membrane lipids: where they are and how they behave. *Nat. Rev. Mol. Cell Biol.* 9:112–124.
13. Frisz, J. F., H. A. Klitzing, ..., M. L. Kraft. 2013. Sphingolipid domains in the plasma membranes of fibroblasts are not enriched with cholesterol. *J. Biol. Chem.* 288:16855–16861.
14. Frisz, J. F., K. Lou, ..., M. L. Kraft. 2013. Direct chemical evidence for sphingolipid domains in the plasma membranes of fibroblasts. *Proc. Natl. Acad. Sci. USA.* 110:E613–E622.
15. Veatch, S. L., and S. L. Keller. 2005. Seeing spots: complex phase behavior in simple membranes. *Biochim. Biophys. Acta.* 1746:172–185.
16. Killian, J. A. 1998. Hydrophobic mismatch between proteins and lipids in membranes. *Biochim. Biophys. Acta.* 1376:401–415.
17. Pabst, G. 2013. Coupling of membrane elasticity and structure to protein function. *In* Advances in Planar Lipid Bilayers and Liposomes, *Vol. 18*. A. Iglic, and C. Kulkarni, editors. Academic Press, London, United Kingdom, p. 282.
18. Binder, W. H., V. Barragan, and F. M. Menger. 2003. Domains and rafts in lipid membranes. *Angew. Chem. Int. Ed. Engl.* 42:5802–5827.
19. Veatch, S. L., and S. L. Keller. 2002. Organization in lipid membranes containing cholesterol. *Phys. Rev. Lett.* 89:268101.
20. Pitcher, 3rd, W. H., S. L. Keller, and W. H. Huestis. 2002. Interaction of nominally soluble proteins with phospholipid monolayers at the air-water interface. *Biochim. Biophys. Acta.* 1564:107–113.
21. McIntosh, T. J., A. Vidal, and S. A. Simon. 2003. Sorting of lipids and transmembrane peptides between detergent-soluble bilayers and detergent-resistant rafts. *Biophys. J.* 85:1656–1666.
22. Gandhavadi, M., D. Allende, ..., T. J. McIntosh. 2002. Structure, composition, and peptide binding properties of detergent soluble bilayers and detergent resistant rafts. *Biophys. J.* 82:1469–1482.
23. Davis, J. H., and M. L. Schmidt. 2014. Critical behavior in DOPC/DPPC/cholesterol mixtures: static ^{2}H NMR line shapes near the critical point. *Biophys. J.* 106:1970–1978.
24. Chiang, Y.-W., Y. Shimoyama, ..., J. H. Freed. 2004. Dynamic molecular structure of DPPC-DLPC-cholesterol ternary lipid system by spin-label electron spin resonance. *Biophys. J.* 87:2483–2496.
25. Ionova, I. V., V. A. Livshits, and D. Marsh. 2012. Phase diagram of ternary cholesterol/palmitoylsphingomyelin/palmitoyloleoyl-phosphatidylcholine mixtures: spin-label EPR study of lipid-raft formation. *Biophys. J.* 102:1856–1865.
26. Tokumasu, F., A. J. Jin, ..., J. A. Dvorak. 2003. Nanoscopic lipid domain dynamics revealed by atomic force microscopy. *Biophys. J.* 84:2609–2618.
27. Mills, T. T., S. Tristram-Nagle, ..., G. W. Feigenson. 2008. Liquid-liquid domains in bilayers detected by wide angle x-ray scattering. *Biophys. J.* 95:682–690.
28. Chen, L., Z. Yu, and P. J. Quinn. 2007. The partition of cholesterol between ordered and fluid bilayers of phosphatidylcholine: a synchrotron x-ray diffraction study. *Biochim. Biophys. Acta.* 1768:2873–2881.
29. Bennett, W. F. D., and D. P. Tieleman. 2013. Computer simulations of lipid membrane domains. *Biochim. Biophys. Acta.* 1828:1765–1776.
30. Akimov, S. A., P. I. Kuzmin, ..., Y. A. Chizmadzhev. 2004. An elastic theory for line tension at a boundary separating two lipid monolayer regions of different thickness. *J. Electroanal. Chem.* 564:13–18.
31. Khelashvili, G., B. Kollmitzer, ..., D. Harries. 2013. Calculating the bending modulus for multicomponent lipid membranes in different thermodynamic phases. *J. Chem. Theory Comput.* 9:3866–3871.
32. Rawicz, W., B. A. Smith, ..., E. Evans. 2008. Elasticity, strength, and water permeability of bilayers that contain raft microdomain-forming lipids. *Biophys. J.* 94:4725–4736.
33. Pabst, G., N. Kučerka, ..., J. Katsaras. 2010. Applications of neutron and x-ray scattering to the study of biologically relevant model membranes. *Chem. Phys. Lipids.* 163:460–479.
34. Pan, J., F. A. Heberle, ..., J. Katsaras. 2013. Using small-angle neutron scattering to detect nanoscopic lipid domains. *Chem. Phys. Lipids.* 170-171:19–32.
35. Heberle, F. A., R. S. Petruzielo, ..., J. Katsaras. 2013. Bilayer thickness mismatch controls domain size in model membranes. *J. Am. Chem. Soc.* 135:6853–6859.

36. Tsamaloukas, A., H. Szadkowska, and H. Heerklotz. 2006. Nonideal mixing in multicomponent lipid/detergent systems. *J. Phys. Condens. Matter.* 18:S1125–S1138.

37. Tayebi, L., Y. Ma, …, A. N. Parikh. 2012. Long-range interlayer alignment of intralayer domains in stacked lipid bilayers. *Nat. Mater.* 11:1074–1080.

38. Karmakar, S., B. R. Sarangi, and V. A. Raghunathan. 2006. Phase behavior of lipid-cholesterol membranes. *Solid State Commun.* 139: 630–634.

39. Pabst, G., M. Rappolt, …, P. Laggner. 2000. Structural information from multilamellar liposomes at full hydration: full q-range fitting with high quality x-ray data. *Phys. Rev. E Stat. Phys. Plasmas Fluids Relat. Interdiscip. Topics.* 62 (3 Pt B):4000–4009.

40. Kučerka, N., J. F. Nagle, …, J. Katsaras. 2008. Lipid bilayer structure determined by the simultaneous analysis of neutron and x-ray scattering data. *Biophys. J.* 95:2356–2367.

41. Heftberger, P., B. Kollmitzer, …, G. Pabst. 2014. Global small-angle x-ray scattering data analysis for multilamellar vesicles: the evolution of the scattering density profile model. *J. Appl. Cryst.* 47:173–180.

42. Hammersley, A. 1997. ESRF Internal Report, ESRF97HA02T, FIT2D: An Introduction and Overview. European Synchrotron Radiation Facility. Grenoble, France.

43. Glatter, O., and O. Kratky. 1982. Small Angle X-Ray Scattering, Vol. 102. Academic Press, London, United Kingdom.

44. Caillé, A. 1972. Notes on the distribution of x-rays in smectics [Remarques sur la diffusion des rayons x dans les smectiques A]. *Acad. Sci. Paris B.* 274:891–893.

45. Zhang, R., R. M. Suter, and J. F. Nagle. 1994. Theory of the structure factor of lipid bilayers. *Phys. Rev. E Stat. Phys. Plasmas Fluids Relat. Interdiscip. Topics.* 50:5047–5060.

46. Pabst, G., R. Koschuch, …, P. Laggner. 2003. Structural analysis of weakly ordered membrane stacks. *J. Appl. Cryst.* 36:1378–1388.

47. Kučerka, N., M.-P. Nieh, and J. Katsaras. 2011. Fluid phase lipid areas and bilayer thicknesses of commonly used phosphatidylcholines as a function of temperature. *Biochim. Biophys. Acta.* 1808:2761–2771.

48. Pan, J., X. Cheng, …, J. Katsaras. 2012. Interactions between ether phospholipids and cholesterol as determined by scattering and molecular dynamics simulations. *J. Phys. Chem. B.* 116:14829–14838.

49. Uppamoochikkal, P., S. Tristram-Nagle, and J. F. Nagle. 2010. Orientation of tie-lines in the phase diagram of DOPC/DPPC/cholesterol model biomembranes. *Langmuir.* 26:17363–17368.

50. Heberle, F. A., J. Wu, …, G. W. Feigenson. 2010. Comparison of three ternary lipid bilayer mixtures: FRET and ESR reveal nanodomains. *Biophys. J.* 99:3309–3318.

51. Koenig, B. W., and K. Gawrisch. 2005. Specific volumes of unsaturated phosphatidylcholines in the liquid crystalline lamellar phase. *Biochim. Biophys. Acta.* 1715:65–70.

52. Greenwood, A. I., S. Tristram-Nagle, and J. F. Nagle. 2006. Partial molecular volumes of lipids and cholesterol. *Chem. Phys. Lipids.* 143:1–10.

53. Davis, J. H., J. J. Clair, and J. Juhasz. 2009. Phase equilibria in DOPC/DPPC-d62/cholesterol mixtures. *Biophys. J.* 96:521–539.

54. Veatch, S. L., O. Soubias, …, K. Gawrisch. 2007. Critical fluctuations in domain-forming lipid mixtures. *Proc. Natl. Acad. Sci. USA.* 104: 17650–17655.

55. Veatch, S. L., I. V. Polozov, …, S. L. Keller. 2004. Liquid domains in vesicles investigated by NMR and fluorescence microscopy. *Biophys. J.* 86:2910–2922.

56. Pan, J., S. Tristram-Nagle, and J. F. Nagle. 2009. Effect of cholesterol on structural and mechanical properties of membranes depends on lipid chain saturation. *Phys. Rev. E Stat. Nonlin. Soft Matter Phys.* 80:021931.

57. McConnell, H. M., and A. Radhakrishnan. 2003. Condensed complexes of cholesterol and phospholipids. *Biochim. Biophys. Acta.* 1610:159–173.

58. Hodzic, A., M. Rappolt, …, G. Pabst. 2008. Differential modulation of membrane structure and fluctuations by plant sterols and cholesterol. *Biophys. J.* 94:3935–3944.

59. Pan, J., T. T. Mills, …, J. F. Nagle. 2008. Cholesterol perturbs lipid bilayers nonuniversally. *Phys. Rev. Lett.* 100:198103.

60. Pabst, G., H. Amenitsch, …, M. Rappolt. 2004. Structure and fluctuations of phosphatidylcholines in the vicinity of the main phase transition. *Phys. Rev. E Stat. Nonlin. Soft Matter Phys.* 70:021908.

61. Pabst, G., J. Katsaras, …, M. Rappolt. 2003. Structure and interactions in the anomalous swelling regime of phospholipid bilayers. *Langmuir.* 19:1716–1722.

62. Parsegian, V. A., R. P. Rand, and D. C. Rau. 1995. Macromolecules and water: probing with osmotic stress. *In* Energetics of Biological Macromolecules, Vol. 259, Methods in Enzymology. M. L. Johnson and G. K. Ackers, editors. Academic Press, London, United Kingdom, pp. 43–94.

63. Buboltz, J. T., C. Bwalya, …, M. Schutzer. 2007. High resolution mapping of phase behavior in a ternary lipid mixture: do lipid-raft phase boundaries depend on sample-prep procedure? *Langmuir.* 23:11968–11971.

64. Mouritsen, O. G., and K. Jørgensen. 1994. Dynamical order and disorder in lipid bilayers. *Chem. Phys. Lipids.* 73:3–25.

65. Honerkamp-Smith, A. R., P. Cicuta, …, S. L. Keller. 2008. Line tensions, correlation lengths, and critical exponents in lipid membranes near critical points. *Biophys. J.* 95:236–246.

66. Honerkamp-Smith, A. R., B. B. Machta, and S. L. Keller. 2012. Experimental observations of dynamic critical phenomena in a lipid membrane. *Phys. Rev. Lett.* 108:265702.

67. Veatch, S. L., P. Cicuta, …, B. Baird. 2008. Critical fluctuations in plasma membrane vesicles. *ACS Chem. Biol.* 3:287–293.

68. Connell, S. D., G. Heath, …, A. Kisil. 2013. Critical point fluctuations in supported lipid membranes. *Faraday Discuss.* 161:91–150.

69. Kollmitzer, B., P. Heftberger, …, G. Pabst. 2013. Monolayer spontaneous curvature of raft-forming membrane lipids. *Soft Matter.* 9:10877–10884.

70. Kučerka, N., J. Katsaras, and J. F. Nagle. 2010. Comparing membrane simulations to scattering experiments: introducing the SIMTOEXP software. *J. Membr. Biol.* 235:43–50.

Supporting Material

In situ determination of structure and fluctuations of coexisting fluid membrane domains

Peter Heftberger,[†,‡] Benjamin Kollmitzer,[†,‡] Alexander A. Rieder,[†,‡] Heinz Amenitsch[§], and Georg Pabst[†,‡] *

[†] *University of Graz, Institute of Molecular Biosciences, Biophysics Division, NAWI Graz, Humboldtstr. 50/III, A-8010 Graz, Austria.*

[‡] *BioTechMed-Graz, Austria.*

[§] *Graz University of Technology, Institute of Inorganic Chemistry, Stremayrgasse 9/IV, A-8010 Graz, Austria.*

[*] Correspondence: georg.pabst@uni-graz.at

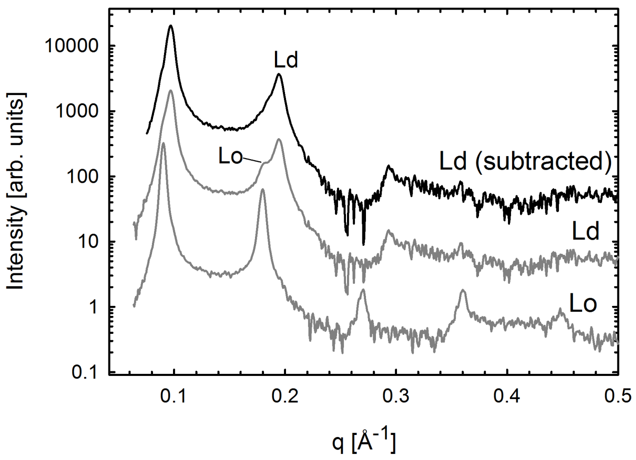

Figure S1: Subtraction of L_o contribution from L_d endpoint of B_{t2}. Scattering intensities are scaled by a constant for clarity. Second order Bragg reflections of L_o and L_d phases are indicated. Because of the overlap of L_o peaks for L_o and L_d endpoints (gray lines), we were able to subtract a fraction of L_o from the L_d endpoint sample. The result is shown in the top scattering pattern (black line).

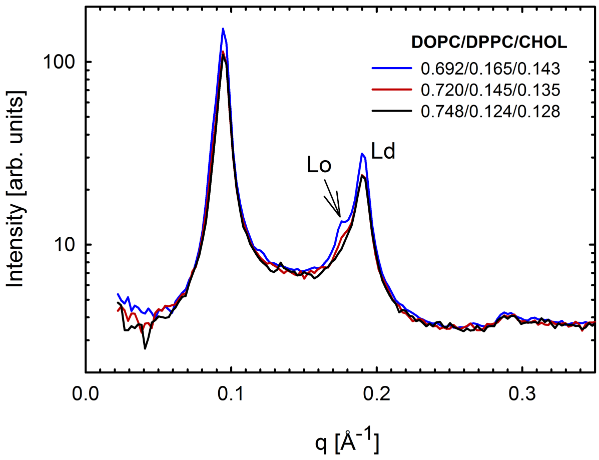

Figure S2: Determination of new L_d tieline endpoint for B_{t2}. Scattering patterns for three different lipid compositions, extending the B_{t2} tieline (1) toward the pure L_d regime (same angle), are shown. Data were recorded on a S3-Micro compact Kratky camera (Hecus X-ray Systems, Graz, Austria) at 15 °C. The second order Bragg peak clearly indicates L_o contamination. No residual L_o was observed for the DOPC/DPPC/CHOL composition 0.748/0.124/0.128 (molar fractions).

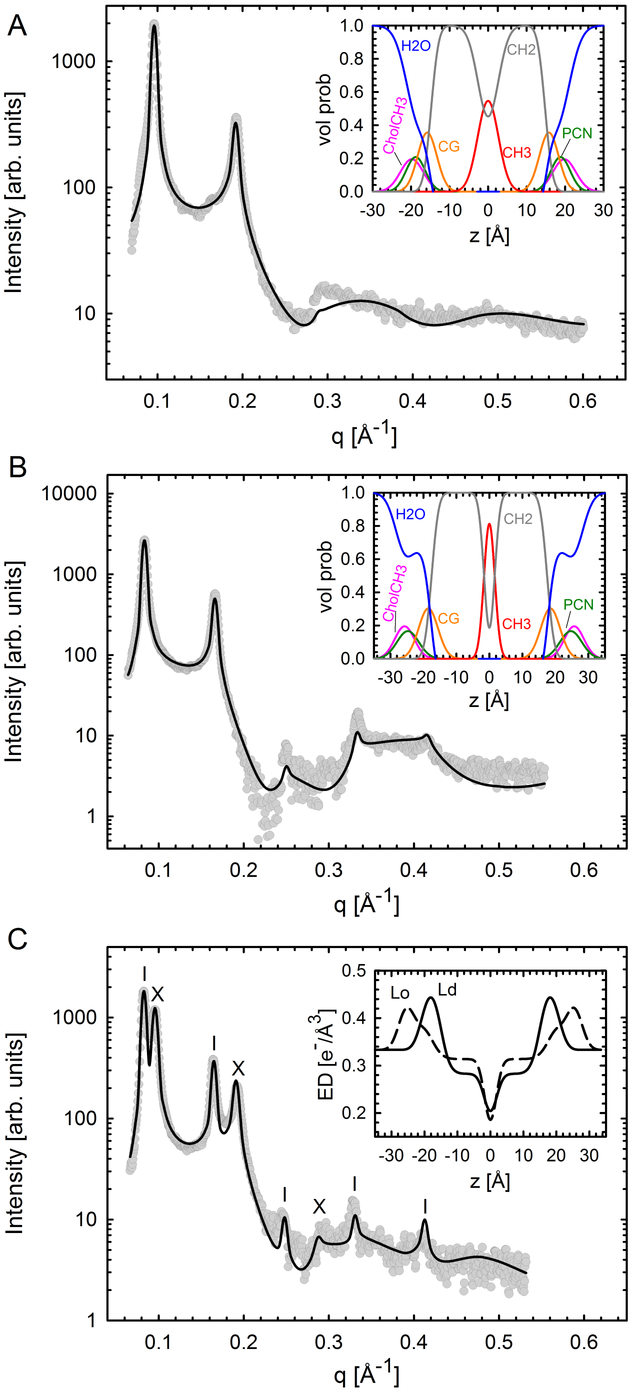

Figure S3: Validation of the global analysis for two coexisting phases for the A_{t2} tieline ($T = 22$ °C). Panels A and B show fits to L_d and L_o endpoint data, respectively. The insets to both panels give the derived volume probability distributions. Panel C shows the best fit to SAXS data at the A_{t2} tieline midpoint. Bragg reflections of L_o and L_d domains are indicated with dashes and crosses, respectively. The corresponding inset gives the ED profiles for L_o and L_d phases.

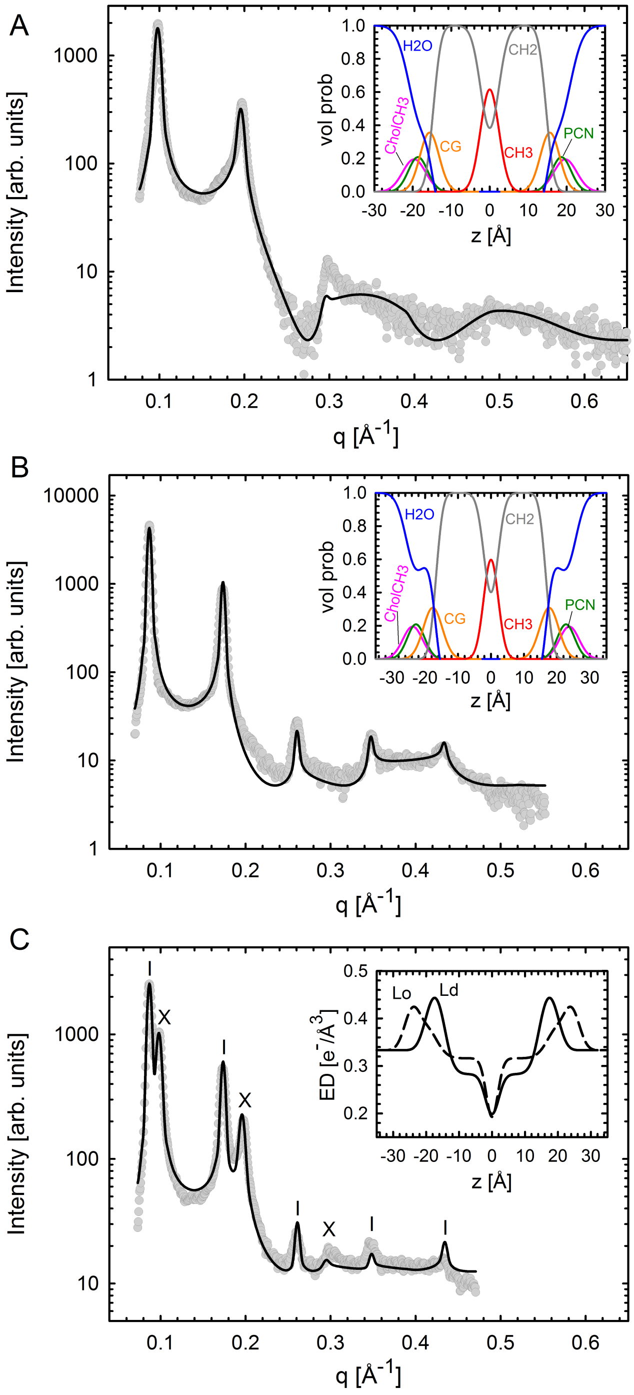

Figure S4: Validation of the global analysis for two coexisting phases for the B_{t1} tieline ($T = 15\,^{\circ}\mathrm{C}$). Panels A and B show fits to L_d and L_o endpoint data, respectively. The insets to both panels give the derived volume probability distributions. Panel C shows the best fit to SAXS data at the B_{t1} tieline midpoint. Bragg reflections of L_o and L_d domains are indicated with dashes and crosses, respectively. The corresponding inset gives the ED profiles for L_o and L_d phases.

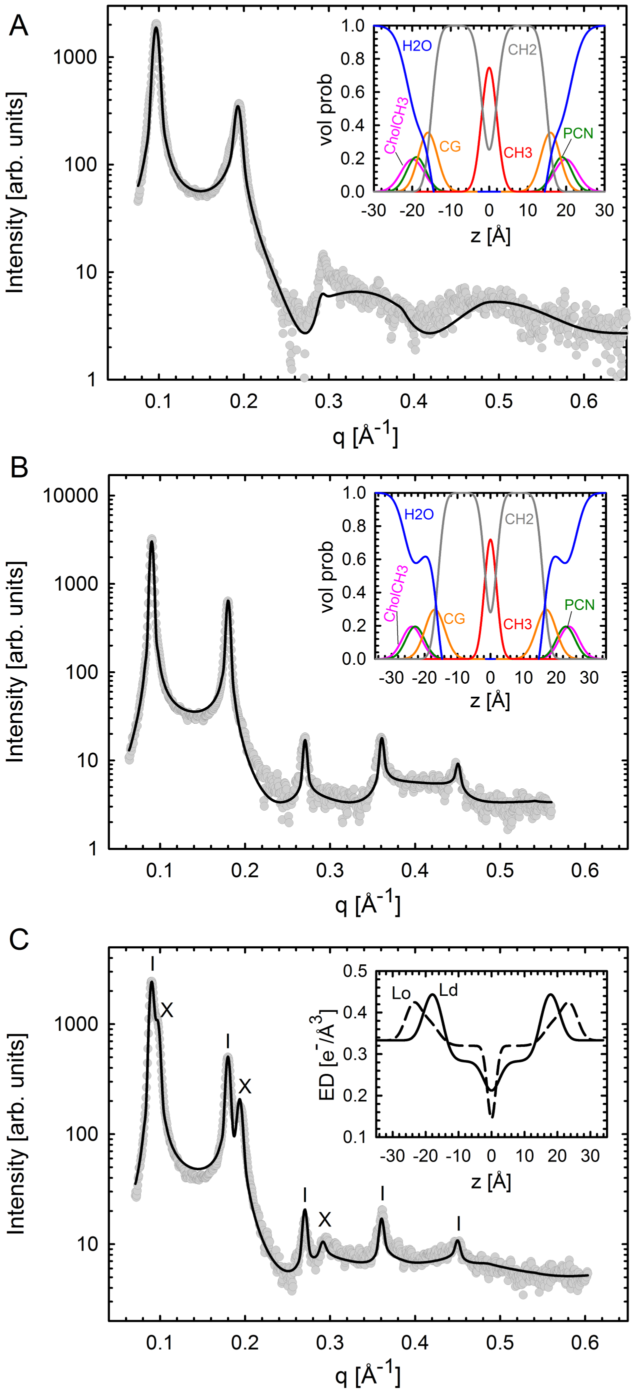

Figure S5: Validation of the global analysis for two coexisting phases for the B_{t2} tieline ($T = 15\,^{\circ}\mathrm{C}$). Panels A and B show fits to L_d and L_o endpoint data, respectively. The insets to both panels give the derived volume probability distributions. Panel C shows the best fit to SAXS data at the B_{t2} tieline midpoint. Bragg reflections of L_o and L_d domains are indicated with dashes and crosses, respectively. The corresponding inset gives the ED profiles for L_o and L_d phases.

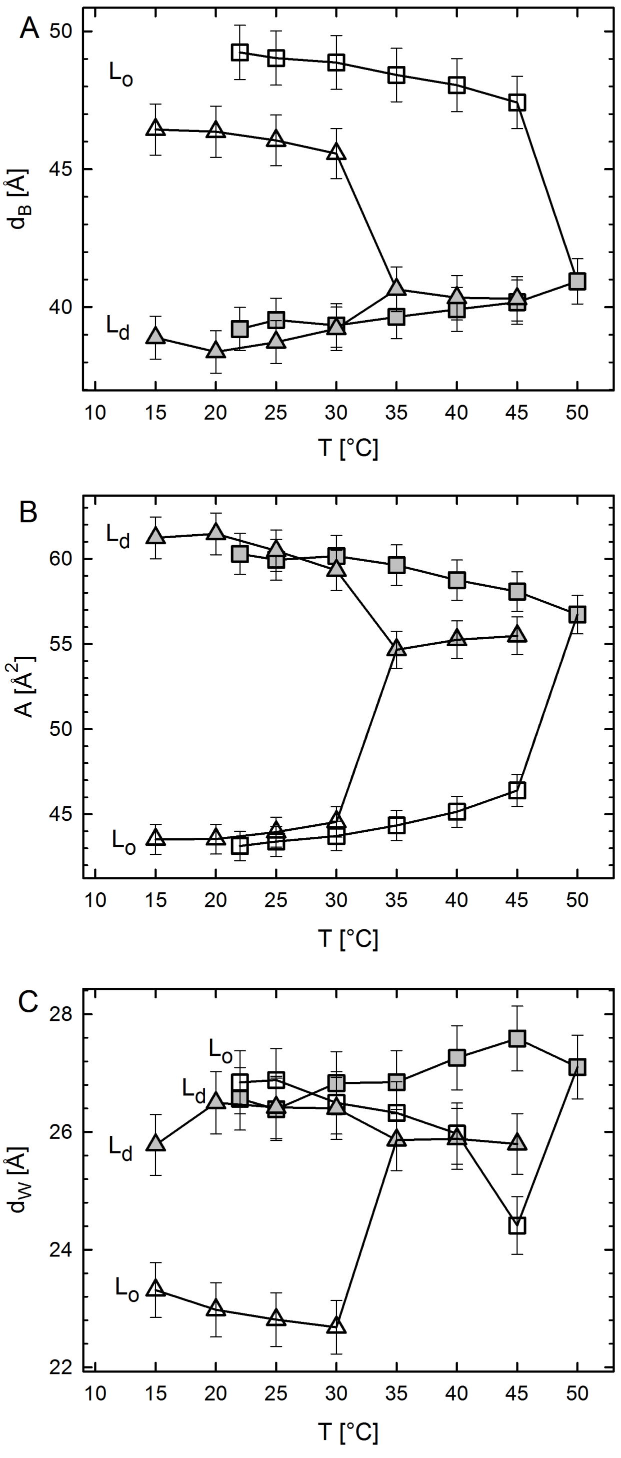

Figure S6: Temperature dependence of structural parameters of coexisting DOPC/DSPC/CHOL (squares) and DOPC/DPPC/CHOL (triangles) domains for $t2$ tieline compositions (Fig. 1, Table S1). Panels A–C show the bilayer thickness, area per lipid, and water layer thickness, respectively for L_d (solid symbols) and L_o (open symbols) domains.

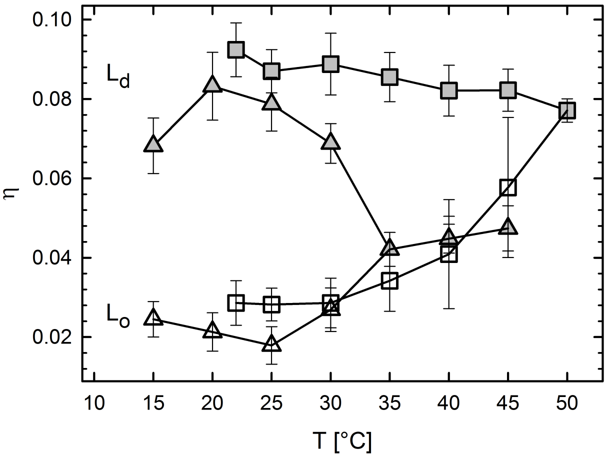

Figure S7: Temperature dependence of bending fluctuations of coexisting DOPC/DSPC/CHOL (squares) and DOPC/DPPC/CHOL (triangles) domains for $t2$ tieline compositions (Fig. 1, Table S1). Solid symbols show results for L_d and open symbols for L_o domains, respectively.

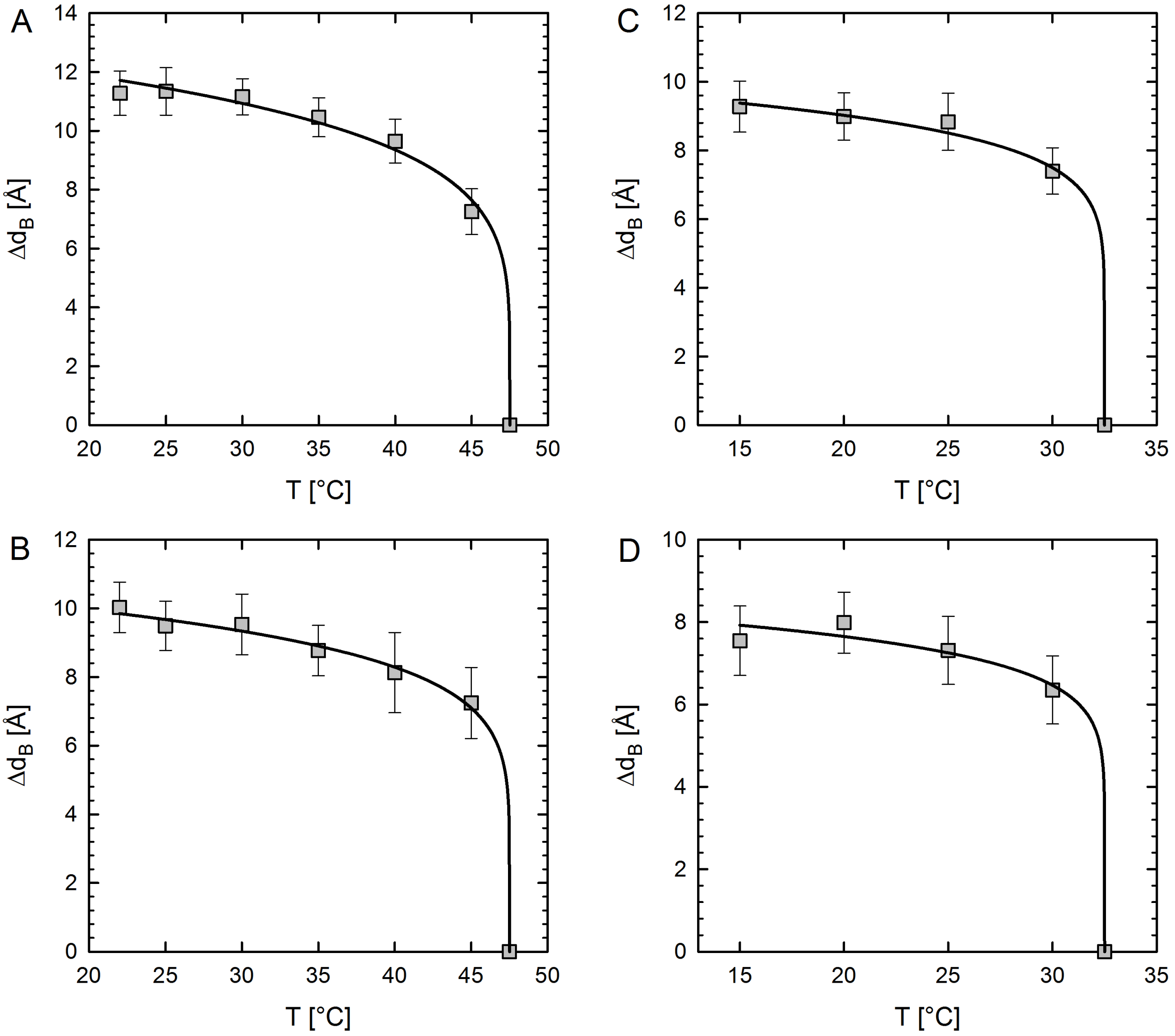

Figure S8: Critical behavior of height difference Δd_B between L_o and L_d domains of tieline midpoint samples A_{t1} (A), A_{t2} (B), B_{t1} (C) and B_{t2} (D). Solid lines correspond to the best fits using $\Delta d_B \propto (T_C - T)^\beta$. T_C was estimated to be 47.5 °C for (A,B) and 32.5 °C for (C,D) yielding the critical exponents $\beta_A = 0.18$, $\beta_B = 0.14$, $\beta_C = 0.115$ and $\beta_D = 0.105$. To evaluate the influence of our choice of T_C, T_C was varied between the extremes of the experimental temperature interval. For $T_C^{min} = 46/31$ °C (for A,B/C,D), we obtained $\beta_A = 0.14$, $\beta_B = 0.11$, $\beta_C = 0.08$ and $\beta_D = 0.08$. For $T_C^{max} = 50/35$ °C (for A,B/C,D), we found $\beta_A = 0.24$, $\beta_B = 0.19$, $\beta_C = 0.16$ and $\beta_D = 0.24$.

Table S1: Lipid compositions (molar fractions) of measured samples according to published compositional phase diagrams (1, 2). Two tielines with a lower (A_{t1}, B_{t1}) and higher (A_{t2}, B_{t2}) cholesterol concentration were studied (see Fig. 1).

		DOPC	DSPC	CHOL
	L_d	0.79	0.09	0.12
A_{t1}	L_o	0.05	0.65	0.30
	L_d/L_o	0.42	0.37	0.21
	L_d	0.74	0.09	0.17
A_{t2}	L_o	0.12	0.56	0.32
	L_d/L_o	0.46	0.30	0.24
		DOPC	DPPC	CHOL
	L_d	0.70	0.23	0.07
B_{t1}	L_o	0.11	0.68	0.21
	L_d/L_o	0.37	0.47	0.16
	L_d	0.66	0.19	0.15
B_{t2}	L_o	0.12	0.58	0.30
	L_d/L_o	0.36	0.41	0.23

Table S2: Structural results and bending fluctuations for the studied tieline endpoints. V_L are the calculated and V_L' the experimentally determined molecular volumes of an effective lipid molecule, including contributions from all three components (for details, see subsequent section). Parameter uncertainties are $< 2\,\%$.

	d_B[Å]	A[Å^2]	d_W[Å]	d_C[Å]	η	V_L[Å^3]	V_L'[Å^3]
A_{t1}-L_d	38.6	62.9	25.8	14.6	0.079	1215	1233
A_{t1}-L_o	48.6	44.2	26.9	18.2	0.047	1075	1063
A_{t2}-L_d	39.1	60.5	26.3	14.8	0.097	1183	1200
A_{t2}-L_d	48.0	44.3	27.4	17.9	0.060	1065	1058
B_{t1}-L_d	38.8	63.5	25.2	14.7	0.082	1231	1227
B_{t1}-L_o	46.6	44.9	25.8	17.4	0.027	1047	1049
B_{t2}-L_d	39.3	60.6	25.6	14.9	0.078	1191	1183
B_{t2}-L_o	45.9	44.0	23.7	17.0	0.028	1010	1015

Lipid volume calculation

The calculated lipid volumes of the ternary mixtures described in Material and Methods, were compared to experimentally determined volumes using the density sound analyzer DSA5000 (Anton Paar, Graz, Austria). Specifically, experiments yielded the partial specific volume (see e.g. (3))

$$v_s = \frac{1}{\rho_0}\left(1 - \frac{\rho_s - \rho_0}{c}\right), \tag{1}$$

where ρ_0 is the density of the solvent, ρ_s density of the solution and c the solute concentration. The experimental volume per molecule is then calculated according to Greenwood et al. as (4):

$$V_L' = \frac{v_s}{N_A}(x_1 M_1 + x_2 M_2 + x_3 M_3), \tag{2}$$

where N_A is Avogadro's number, x_i molar fraction and M_i the molecular weights.

Alternatively, volumes can be calculated by (5)

$$V_L(T) = V_H + n_{CH} V_{CH}(T) + n_{CH2} V_{CH2}(T) + n_{CH3} V_{CH3}(T), \tag{3}$$

where the head group volume V_H is kept fixed at 319 Å^3. V_{CH}, V_{CH2}, V_{CH3} are the segmental volumes of methine-, methylene-, and methyl groups and n_{CH}, n_{CH2}, n_{CH3} are the number of CH, CH_2, CH_3 groups per lipid molecule, respectively. A detailed description of the temperature dependence can be found in (5). Experimental and calculated molecular volumes were found to agree within experimental uncertainties (Table S2).

Estimation of temperature dependent compositional changes

Assuming that the inclination of tielines remains constant with temperature, tieline endpoints can be estimated using the lever rule. Specifically, we determined the distances between tieline midpoints and tieline endpoints $\overline{Ld}$, $\overline{Lo}$ using

$$c_{Ld}(T) = \frac{\overline{Lo}(T)}{\overline{tl}(T)} \tag{4}$$

$$c_{Lo}(T) = 1 - c_{Ld}(T) = \frac{\overline{Ld}(T)}{\overline{tl}(T)}, \tag{5}$$

where $\overline{tl}(T)$ is the overall tieline length. $\overline{tl}$ was estimated to change with temperature in proportion to the L_o fraction. Arbitrary functions were used to fit $c_{Lo}(T)$ data. Results are shown in Fig. S9. Estimates reproduce the asymmetric closing-in of the phase boundaries reported previously (6), which originate here from the measured increase of the L_d phase fraction.

Uppamoochikkal et al. (1) reported a small but significant increase of the tieline inclination angle with temperature. Therefore we checked, how this would influence our structural results. Note that this concerns only the area per lipid A, as results for all other parameter do not depend on knowing the specific lipid compositions of L_o/L_d domains. Hence, we tested several functions for the temperature behavior of the tielines, without finding any effects on the relative changes of A. Changes were observed for absolute values of A at the higher temperatures. However, they remained small, i.e. within $\pm 3\,\%$.

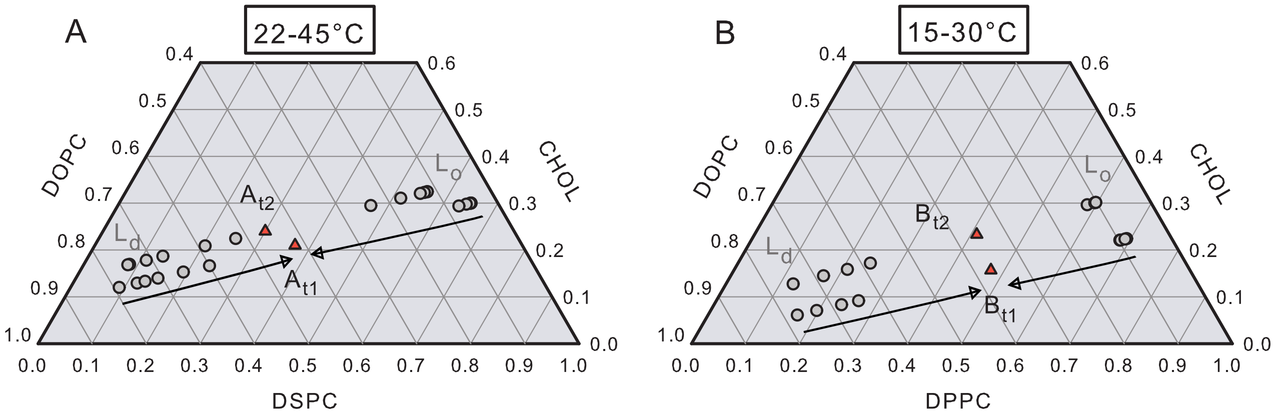

Figure S9: Estimated temperature dependence of tieline endpoints for DOPC/DSPC/CHOL (panel A) and DOPC/DPPC/CHOL (panel B). Triangles represent starting points at the tieline midpoints. Circles indicate the corresponding estimated tieline endpoints, which shift toward the midpoints as temperature is increased. The temperature increment was 5 ℃ only the first temperature step for DOPC/DSPC/CHOL was smaller (3 ℃). Note that L_d endpoints approach tieline midpoints more rapidly than L_o endpoints.

Supporting References

1. Uppamoochikkal, P., S. Tristram-Nagle, and J. F. Nagle, 2010. Orientation of tie-lines in the phase diagram of DOPC:DPPC:cholesterol model biomembranes. *Langmuir* 26:17363–17368.

2. Heberle, F. A., J. Wu, S. L. Goh, R. S. Petruzielo, and G. W. Feigenson, 2010. Comparison of three ternary lipid bilayer mixtures: FRET and ESR reveal nanodomains. *Biophys. J* . 99:3309–3318.

3. Hodzic, A., M. Rappolt, H. Amenitsch, P. Laggner, and G. Pabst, 2008. Differential modulation of membrane structure and fluctuations by plant sterols and cholesterol. *Biophys. J.* 94:3935–3944.

4. Greenwood, A. I., S. Tristram-Nagle, and J. F. Nagle, 2006. Partial molecular volumes of lipids and cholesterol. *Chem. Phys. Lipids* 143:1–10.

5. Koenig, B. W., and K. Gawrisch, 2005. Specific volumes of unsaturated phosphatidylcholines in the liquid crystalline lamellar phase. *Biochim. Biophys. Acta.* 1715:65–70.

6. Buboltz, J. T., C. Bwalya, K. Williams, and M. Schutzer, 2007. High resolution mapping of phase behavior in a ternary lipid mixture: Do lipid-raft phase boundaries depend on sample-prep procedure? *arXiv:0706.1374 [physics]* arXiv: 0706.1374.
